# A systematic review of MHPSS interventions targeting non-clinical Arabic-speaking refugees and/or displaced populations in the MENA region

**DOI:** 10.1136/bmjgh-2025-021128

**Published:** 2026-07-30

**Authors:** Luma Bashmi, Derrick Silove, Carol Brayne, Isla Kuhn, Eolene Boyd

**Affiliations:** 1 Department of Psychiatry, University of Cambridge School of Clinical Medicine, Cambridge, UK; 2 Cambridge Public Health, University of Cambridge School of Clinical Medicine, Cambridge, UK; 3 University of New South Wales, Sydney, New South Wales, Australia; 4 Medical Library, School of Clinical Medicine, University of Cambridge, Cambridge, UK

**Keywords:** Mental Health & Psychiatry, Public Health, Global Health, Systematic review, Delivery of Health Care

## Abstract

**Background:**

Arabic-speaking refugees and displaced populations in the Middle East and North Africa (MENA) face ongoing adversity that negatively impacts their mental health. Despite increasing mental health needs, there is no published systematic review evaluating non-clinical mental health and psychosocial support (MHPSS) interventions in the MENA region for this population, particularly through a culturally grounded framework.

**Methods:**

We conducted a systematic review of MHPSS interventions published from 2011 to 2022 targeting non-clinical Arabic-speaking adult refugees and/or displaced persons in MENA countries. Following Preferred Reporting Items for Systematic Reviews and Meta-Analyses (PRISMA) 2020 guidelines and PROSPERO registration (CRD42023421057), we searched ten databases and grey literature sources. Objectives were to identify, appraise and synthesise eligible MHPSS interventions and assess their alignment with the Integrative Complexity (IC)-Adaptation and Development After Persecution and Trauma (ADAPT) framework. Studies were appraised using four validated quality assessment tools and a novel tool assessing links to the IC-ADAPT framework, which integrates an eco-psycho-social model (ADAPT) and a cognitive-interactionist model (IC). A subset of ten top-rated interventions was synthesised for IC-ADAPT alignment.

**Results:**

Thirty-eight studies met the inclusion criteria. Most interventions were conducted in Lebanon or Jordan, group-based and delivered by non-specialist facilitators in community settings. Parenting, resilience-building and trauma-focused therapies were the most common modalities. Most studies prioritised feasibility and effectiveness over efficacy. While none explicitly applied IC-ADAPT, several implicitly aligned with its eco-psychosocial systems (eg, bonds/networks, safety/security and roles/identities) and IC (eg, increased ‘differentiation’), reporting reduced harsh parenting and improved reflective capacity. Culturally adapted interventions that promoted complex thinking, relational healing and community participation were linked with more favourable outcomes.

**Conclusion:**

This review highlights the value of culturally sensitive, non-clinical MHPSS interventions in enhancing mental health outcomes for Arabic-speaking refugee populations in MENA. Programmes grounded in eco-psychosocial and cognitive complexity frameworks—such as IC-ADAPT—offer effective, scalable strategies for addressing refugee mental health. Future work should embed these models explicitly to optimise intervention design, relevance and impact.

**PROSPERO registration number:**

CRD42023421057.

**WHAT IS ALREADY KNOWN ON THIS TOPIC**

- Arabic-speaking refugees in the Middle East and North Africa (MENA) region face persistent mental health challenges due to displacement and conflict, yet access to culturally appropriate, non-clinical mental health and psychosocial support (MHPSS) interventions remains limited.
- Existing reviews have focused on clinical interventions or populations outside the region, and few have applied an integrated theoretical framework. There was a need to evaluate the quality and relevance of MHPSS interventions tailored to this population.

**WHAT THIS STUDY ADDS**

- This review identifies feasible, effective and implementable MHPSS interventions delivered to non-clinical populations across MENA, many of which implicitly align with the Integrative Complexity-Adaptation and Development After Persecution and Trauma (IC-ADAPT) framework despite not referencing it explicitly.
- It highlights which ADAPT systems - such as strengthening social bonds/networks and roles/identities - are most impactful and how they intersect with cognitive-emotional processing to foster adaptive responses in displaced communities.

**HOW THIS STUDY MIGHT AFFECT RESEARCH, PRACTICE OR POLICY**

- This study supports using IC-ADAPT as a guiding framework for developing and evaluating culturally grounded MHPSS interventions. It underscores the importance of addressing structural and relational determinants of distress and offers practical guidance for scaling interventions through non-specialist delivery. Findings can inform policy efforts to embed MHPSS into broader community and humanitarian systems in the region.

## Background

 The number of forcibly displaced people due to conflict, human rights violations, persecution and other disasters reached “unprecedented heights” in 2026, leaving an estimated 122 million refugees, asylum seekers and internally displaced people in need of humanitarian assistance.^1^ According to the United Nations High Commissioner for Refugees (UNHCR), one of the largest displaced populations in the world comes from Syria, with more than 13 million displaced Syrians (7.2 million internally displaced, 6.4 million registered refugees).^2^ Over 70% of displaced people are hosted in low and middle-income countries.^3^ Lebanon hosts the largest number of refugees per capita in the world, with an estimated 1.5 million Syrian refugees and a ratio of 1 refugee per four Lebanese nationals.^4^ Jordan follows as the second largest host of refugees per capita in the Middle East and North Africa (MENA) and fifth globally, with an estimated 1.3 million Syrian refugees, of which over 700 000 are registered.^5 6^ The number of displaced Palestinians follows closely with approximately six million, with both Jordan and Lebanon hosting some of the largest percentages of Palestinian refugees globally.^7^

Arabic-speaking refugees have experienced enduring trauma and related stressors that can have a detrimental impact on their mental health.^8 9^ These include warfare, displacement and resettlement, economic crisis, the COVID-19 pandemic, the Syrian civil war, Beirut port explosion and most recently the Israeli attacks on Gaza and Lebanon in 2023–2026.^4 8^ Traumatic experiences in refugees’ home countries can have a detrimental impact on their mental health, further exacerbated by post-migration stressors including discrimination, lack of access to healthcare and affordable housing, anti-immigration policies, unemployment and inability to make a livelihood.^8–12^

These post-migration stressors not only lead to poor refugee mental health but can also negatively impact refugees’ capacity to adapt to their host countries, reducing socioeconomic equity, inclusion and social and emotional learning.^10–12^ Although studies on the impact of the ongoing crises in the MENA region on Arabic-speaking refugees’ mental health are limited, the effect is expected to be devastating and long-term if sustainable mental health systems are not accessible.^13^ However, most models that explore the impact of trauma on refugee populations adopt a constrained, clinical-focused approach by assessing the prevalence of psychopathology, specifically post-traumatic stress disorder (PTSD), with some using assessments that are not validated in the Arabic-speaking refugee context.^8 9 14^ Further studies have shown that not all refugees exposed to trauma are likely to develop psychopathology but many may experience high rates of psychological distress.^9 13 15^

This suggests that these mental health models may not be generalisable to all populations, underscoring the need for a culturally sensitive approach to understanding how people across cultures experience and express suffering and psychological distress, how they explain illness and misfortune, how they seek help and how intergenerational trauma impacts these nuances.^16–18^ Known as *cultural idioms of distress*, this concept aligns with the broader movement away from strictly clinical models that are limited in scope and conceptualisation and affirmation of community knowledge systems and cultural healing practices in the understanding of distress and its potentially varied cultural expressions.^16 19 20^ Furthermore, although conflicts in the MENA region may temporarily ease with ceasefires, long-term stability remains uncertain. Political shifts, such as changes in leadership, can influence the prospects of displaced populations considering a return to their home countries.^21^ Large-scale repatriation often depends on the restoration of sustainable and secure conditions, is typically slow and shaped by political, economic and humanitarian factors.^22^

Host countries can play a key role in ensuring a safe, dignified and voluntary return for refugees.^23^ Meanwhile, for refugees to feel protected, included and their strengths identified and supported in ways that enable them to contribute to society, host countries must provide effective support to enhance their mental health based on an accurate understanding and longitudinal assessment of these refugees’ experiences accessed through community engagement, coupled with culturally sensitive, evidence-based mental health and psychosocial support (MHPSS) interventions. For example, programmes or practices that directly or indirectly enhance mental health and demonstrate evidence of effectiveness in producing results and improving outcomes when implemented.^24^

In the past few decades, multiple studies have explored the relevance of trauma-based models in explaining traumatic experiences, including how these models are used to frame MHPSS interventions. Recently, the integration of an interactionist cognitive processing model, Integrative Complexity (IC), and an eco-psychosocial model, Adaptation and Development After Persecution and Trauma (ADAPT), into one analytical framework has presented a promising approach in the field of refugee mental health and public mental health promotion.^15^ The combined framework represents an eco-psychosocial approach to integrating individual and group engagement with difference and disagreement and adaptive and maladaptive individual and collective responses to societal crises.^25–27^

IC, an interactionist cognitive processing model, features an empirical measurement frame focusing on the absence or increasing presence of *differentiation* and *integration* to explore how people process and structure their thinkin*g*.^28–31^ Tetlock *et al* explain this as “*How people think depends in part on why people think*”, (p.640).^32^ Differentiation refers to the recognition of different dimensions and perspectives, and integration emerges when complex connections are made between these different dimensions and perspectives. Integration includes structured discussions where group members are exposed to differing opinions (ie, differentiation) and are actively supported to discuss them (ie, integration), for example, moving from “*we’re right, you’re wrong*” to “*I disagree with you, but hear your view*”.^31 33^ Arguably both trait and state-like, low IC involves rigid, categorical thinking that can be associated with conflict and violence, while high IC involves flexible thinking associated with cooperation despite difference and disagreement.^34 35^ In a study feasibility testing an intervention among Muslim and practitioner (eg, social worker, police) communities in Scotland to increase IC and collaboration across communities, post-intervention insights included participants finding more commonality between Scottish and Islamic values, recognising issues that are common to both communities rather than just their own, having tools to better engage with different perspectives, increasing self-regulation and resilience and being more positive about hearing out-group views.^29^

The ADAPT model identifies five interrelating eco-psycho-social systems that support public mental health in stable societies: (1) Safety/security, (2) Interpersonal bonds and networks, (3) Justice, (4) Identities and roles and (5) Existential meaning. Experiencing one or more of these systems as disrupted or broken over prolonged periods, as refugees often experience, can lead to maladaptive reactions with adverse consequences inter-personally, familially and communally.^27 36^ For example, war and conflict can lead to mass migration, causing the breaking of bonds/networks (system II) between families, friends and communities. The loss of bonds/networks has a detrimental impact on individuals’ health and well-being, whether for those who are left behind or move to foreign and potentially hostile settings.^14 37^

The IC-ADAPT framework is infinitely adaptable to culture and context. While the combined framework is relatively new, it has been discussed and presented in several contexts more recently and both models alone are well-established.^11 12 38^ The IC model with empirical coding frame has robust research supporting its reliability and validity spanning over 40 years.^11 28–31 33 34 39–43^ The ADAPT model is one of the few models developed specifically with and for refugees and has been reported for over two decades as reliably helpful to refugees across contexts and populations seeking to understand their experiences.^14 15 27 36 37 44–46^ This systematic review is the first exploration of the IC-ADAPT framework’s relevance to Arabic-speaking refugees and displaced populations in the MENA region.

This review is both timely and essential, given four key challenges in delivering MHPSS interventions in this context. First, no published systematic review has examined interventions targeting Arabic-speaking refugees and displaced populations in MENA affected by humanitarian crises, despite them being one of the largest displaced population.^47 48^

Second, MENA refugees have endured prolonged violence and conflict over generations, increasing the risk of transgenerational trauma.^49^ They have also faced neglect from the global community following the Ukraine war, as international aid and resettlement priorities shifted. For Syrian refugees, this has exacerbated existing cultural challenges in host countries, where they often face legal and social barriers to integration, particularly in neighbouring MENA countries that do not grant them formal refugee status.^16^

Third, most refugee-focused interventions rely on clinical mental health models that may prioritise diagnosing and reducing symptoms without considering how refugees understand their experiences or how crisis events impact their relationships and eco-psycho-social systems, as outlined in the ADAPT model. Cultural validity in assessment methods and interpretations is also often lacking.^8 9 16 20 24 37 44 50–52^

Finally, inconsistencies in MHPSS interventions - spanning terminology, methodology, design, evaluation and implementation - make outcome comparisons difficult. Transferring interventions across settings presents further challenges due to differences in infrastructure for evaluation and follow-up, stigma, meaning making and community practices.^24 53 54^

### Purpose and objectives

This systematic review aims to (1) Identify existing MHPSS interventions in the MENA region targeting non-clinical adult MENA refugees and displaced populations and (2) Assess studies across four quality assessment (QA) tools and a newly designed tool: (1) Feasibility and acceptability, (2) Efficacy and/or effectiveness, (3) Implementation, (4) Risk of bias assessment and (5) Evaluate the extent to which effective interventions’ variables of change link to the IC-ADAPT factors.

This systematic review aims to answer the following research questions:

1. What MHPSS studies have been conducted and interventions tested in MENA host countries with non-clinical adult refugees/displaced populations from the MENA region?
  - What interventions have been implemented in Lebanon and Jordan since 2011?
  - What are their study designs, and which designs are the most common?
  - Do any target females only, males only or both genders specifically?
  - What are the variables of change?
2. Within the interventions that fit the inclusion criteria:
  - Which interventions are evidence-based and how are they defining ‘evidence-based’?
  - How are they evaluated?
  - Which interventions are effective?
  - How did these interventions define and evaluate effectiveness?
  - Of the interventions that are deemed effective, are there any that link with the IC-ADAPT framework?

## Methods

### Protocol and registration

A systematic review was reported in line with the standards of the Preferred Reporting Items for Systematic Reviews and Meta-Analyses (PRISMA) 2020 guidelines.^55^ The systematic review protocol was registered on PROSPERO (ID: CRD42023421057).

### Literature search

From 1 to 12 November 2022, searches were conducted using relevant keywords across four categories (1) Population: refugee, asylum seeker, displaced people, (2) Geography: MENA region, (3) Intervention and (4) IC-ADAPT framework, subject headings (controlled vocabularies) and search syntax, appropriate to each resource in MEDLINE (Ovid), PsycINFO (Ebsco), Global Health (Ebsco), ASSIA (Proquest), Sociological Abstracts (Proquest), ProQuest Dissertations and Theses Abstracting and Indexing Service (PQDT), Scopus, Web of Science Core Collection and WHO Global Index Medicus, Eastern Mediterranean.

Searches were run without limits on language or publication type across databases and grey literature. As the systematic review focused on Arabic-speaking refugees and host communities who were impacted by the Syrian civil war that started in 2011, studies published before 2011 were not included. The search strategy was developed in collaboration with one of the authors (IK). It was translated for the various databases and run across all databases, extracted and deduplicated by IK. A detailed search strategy is described in online supplemental appendix A.

### Eligibility criteria and search strategy

Eligible publications were limited to those reporting on MHPSS interventions within the MENA region targeting non-clinical adult MENA Arabic-speaking refugees and/or displaced populations experiencing grief or mental distress, and were primary quantitative, qualitative or mixed-methods interventions using any of these designs: analytic (experimental/intervention) study design such as randomised controlled trials (RCTs), non-RCTs, cross-over trials, descriptive study design (case reports/studies) and qualitative methodology that must report on a specific intervention and subsequent outcome measure(s).

Eligible participant nationality and intervention locations included Syria, Lebanon, Palestine, Iraq, Yemen, Jordan, Turkey, Egypt, Bahrain, Saudi Arabia, Qatar, United Arab Emirates, Kuwait or Oman. MHPSS interventions were defined as any type of local/outside support aiming to protect/promote directly or indirectly psychosocial well-being or prevent or reduce symptoms of mental distress.

Studies were excluded if: only clinical populations were targeted, sample size was <10, participants were <18 or >65 years old, published in languages other than English or Arabic, systematic reviews/meta-analyses, no outcome measures or insufficient outcome measures relating to the intervention were provided and no full text was available.

We defined non-clinical populations as those experiencing psychological distress without a formal psychiatric diagnosis (eg, no confirmed PTSD or Major Depressive Disorder diagnosis). Clinical populations were defined as those with a *clinical diagnosis* of PTSD or severe depression. It was important to include interventions targeting clinical populations, provided the target population also included non-clinical populations (ie, those with no clinical presence of PTSD, depression or similar mental illness).

We did not exclude studies that were held in *clinical* settings (eg, healthcare centre, clinic) or were facilitated by *clinicians* (eg, psychiatrists, psychologists, therapists, mental health experts). We found it important to compare studies where facilitators were specialists versus non-specialists (ie, peer refugees or non-mental health workers). The type of facilitator is described in study characteristics and assessed under the implementation feasibility tool.

### Data extraction and synthesis

Article review, selection and data extraction was done by two authors (LB and EB). After removing duplicate articles on Endnote (Clarivate, Philadelphia), the authors independently conducted a blind screening of titles and abstracts from 100 eligible papers using Rayyan (Rayyan.ai) to ensure screening was aligned and risk of bias was reduced. Once alignment was achieved, the remaining 2711 papers were screened by LB first, and screened papers LB considered fit or may be fitting (“Yes” and “Maybe” piles) were then screened by EB. Next, a full review of each relevant manuscript was conducted independently by each screener, yielding the final selection of articles for this study. Ninety-five studies required discussion and resolution between the two authors.

Data from the eligible studies were extracted into an extraction sheet manually and with the assistance of an online machine learning tool, Elicit AI, prompted to select data relevant to meet the review objectives (country, setting, study design, intervention type and duration, Inter-Agency Standing Committee (IASC) pyramid layer, cohort characteristics, intervention type, outcomes and mechanisms of change).

#### Quality assessment

Full texts were critically appraised across five scoring tools with a total of 65 criteria, including four QA tools and a newly designed tool specific for IC-ADAPT factors extraction and analysis. Scoring alignment between authors was then finalised. Any discrepancies were resolved by discussion.

The selected four QA tools were shown to be the most valid and reliable in their field: (1) Feasibility and acceptability (Eldridge *et al*),^56^ (2) Efficacy and/or effectiveness (RITES, 2017),^57^ (3) Implementation (ImpRess, 2016)^58^ and (4) Quality assessment for diverse studies (QuADS, 2021)^59^. See online supplemental appendix B for the rationale for selection and scoring guidelines. Scoring alignment between authors was then finalised. Discrepancies in the scoring of 11 studies were resolved by discussion.

### Data analysis

Due to the heterogenous nature of the studies and research designs, a narrative synthesis was conducted following the Guidance on the Conduct of Narrative Synthesis in Systematic Reviews by Popay *et al*.^60^ Results are presented in three sections to align with the research objectives: (1) Study characteristics, (2) Feasibility, effectiveness/effectiveness, implementation and quality of included studies and (3) Subgroup analysis of studies deemed effective synthesised for any links with the IC-ADAPT factors.

#### Study appraisal process

**Figure 1:**
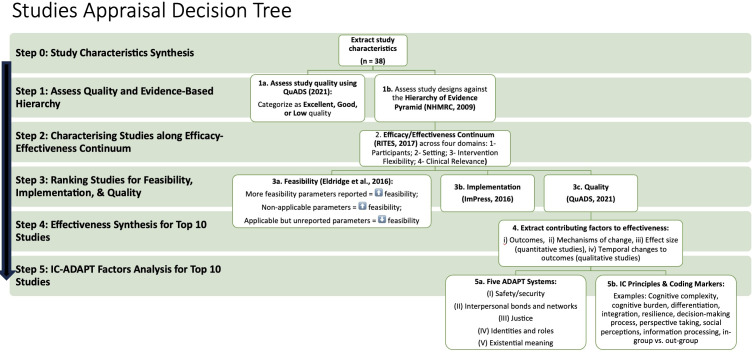
Decision tree for appraisal process of included studies (n=38). ADAPT, Adaptation and Development After Persecution and Trauma; IC, Integrative Complexity; QuADS, Quality assessment for diverse studies; RITES, Rating of Included Trials on the Efficacy–Effectiveness Spectrum.

Study characteristics were synthesised and descriptively summarised to provide an overview of the literature. Full texts were then taken through a five-step appraisal process (figure 1). First, studies were assessed for quality (QuADS, 2021) and categorised as *excellent*, *good* or *low* quality based on the tool scoring guidelines. To determine which studies were deemed ‘effective’, that is, evidence-based, study designs were assessed for their evidence-based approach against the National Health Service (NHS) Hierarchy of Evidence pyramid (online supplemental appendix C). A study design higher up the hierarchy was considered more rigorous in its methodology and hence more likely to minimise the effect of bias on the study results.^61^

Second, studies were characterised along the RITES efficacy/effectiveness continuum via four domains: (1) Participants, (2) Setting, (3) Intervention Flexibility and (4) Clinical Relevance. Lower scores indicated a strong emphasis on efficacy (scores 1 or 2), and higher scores indicated a strong emphasis on effectiveness (scores 4 or 5). A score of 3 indicated a balanced emphasis on both efficacy and effectiveness.

Third, they were ranked to identify the top ten studies that were most feasible (Eldridge *et al* parameters), implementable (ImpRess) and high-quality (QuADS) based on the relevant QA tools’ total scores. As the feasibility tool did not offer scoring guidelines, the authors determined that the greater the number of feasibility criteria reported, the more feasible the study would be deemed. Each criterion was categorised as applicable and reported, non-applicable or applicable but unreported (no information). Studies which had some parameters that were non-applicable (eg, follow-up rates were not reported as the study methods were focus groups) were deemed more feasible than studies where parameters were applicable but unreported.

#### Analysis of subsets

**Figure 2:**
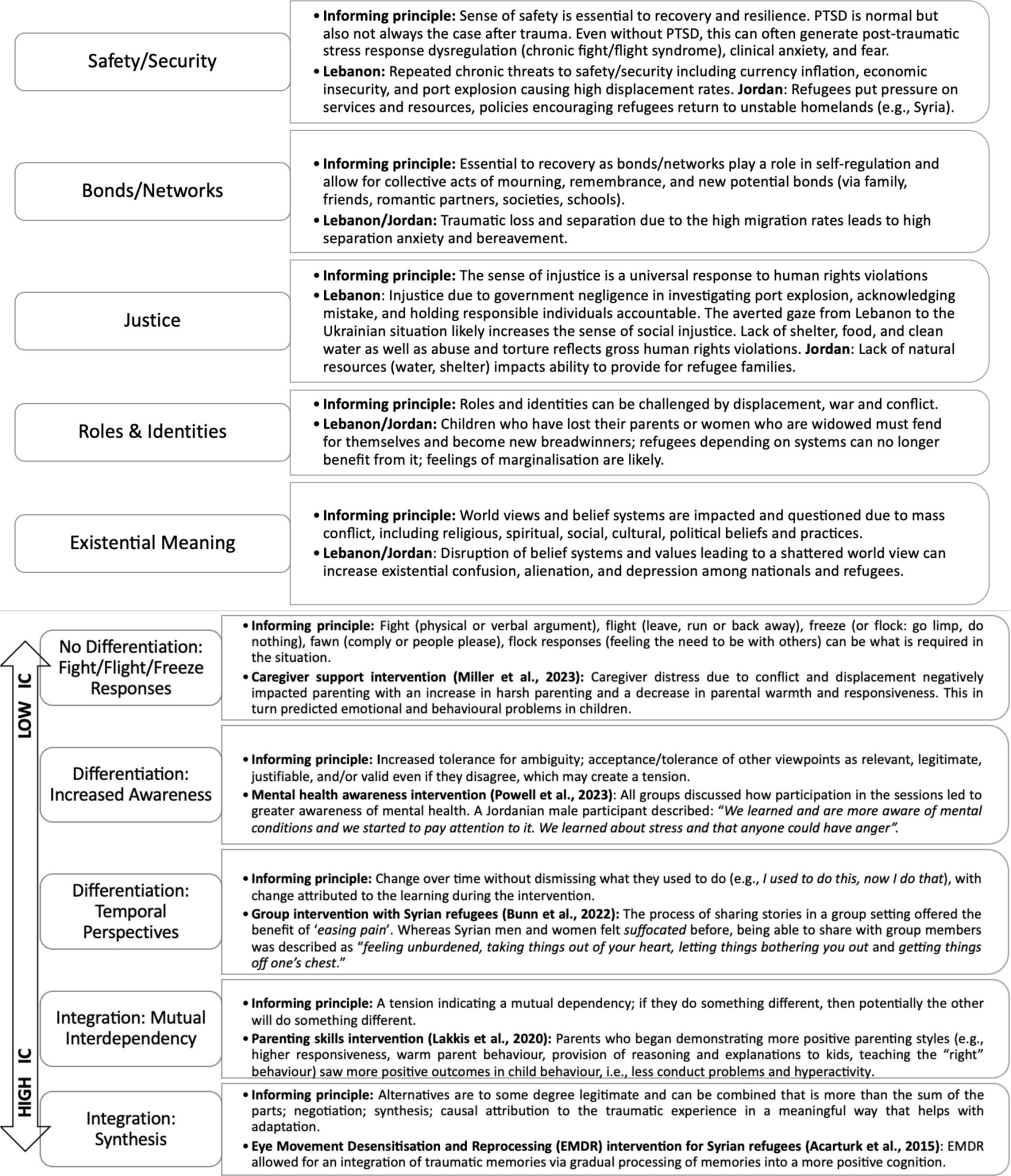
Integrative Complexity (IC)-Adaptation and Development After Persecution and Trauma (ADAPT) Framework: Five ADAPT Systems Applied to the Lebanese and Jordanian Contexts (Informing principles taken from Silove; Tay *et al*) and IC Principles and Coding Markers (all assessments based on the IC coding manual, Bake-Brown). EMDR, Eye Movement Desensitisation and Reprocessing; IC, Integrative Complexity; PTSD, posttraumatic stress disorder.

Fourth, the top ten studies were then synthesised to understand how they defined and evaluated effectiveness by identifying the contributing factors to effect, including outcomes, mechanism(s) of change and effect size for quantitative studies or temporal changes to outcomes for qualitative studies. Finally, they were assessed to determine whether any study characteristics (eg, intervention type, mechanism of change) link to one or more of the IC-ADAPT factors, for example, the five ADAPT systems or any IC principles and coding markers. See figure 2 for examples of IC-ADAPT factors analysis and online supplemental appendices for the IC-ADAPT synthesis tool.

### Patient and public involvement

Patients and the public were not involved in this systematic review as it relied exclusively on the analysis of previously published articles.

## Results

**Figure 3:**
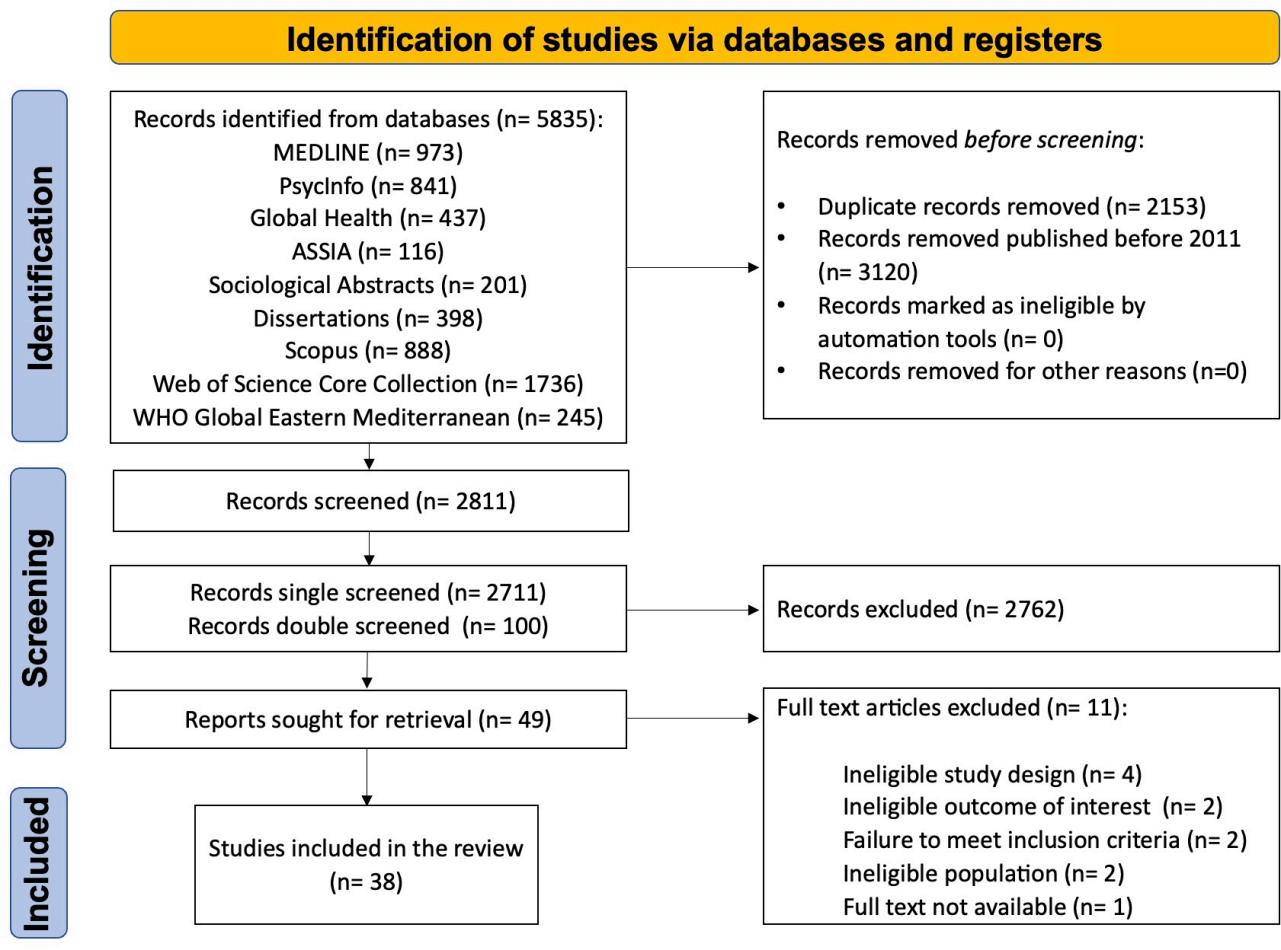
Preferred Reporting Items for Systematic Reviews and Meta-Analyses (PRISMA) Flow Diagram of Systematic Review Study Selection.

Initial database searches yielded 5835 articles (online supplemental appendix A). After removing duplicates, the titles and abstracts of 2811 articles were screened by abstract only (2711 by LB; 100 by EB and LB). The full texts of 49 articles were reviewed by LB and EB, of which 38 articles were included in this review (figure 3). Twenty-four studies were published between 2020–2023, and the remaining studies were published between 2015–2019 (n=14).

### Study characteristics

#### Study setting and population

Study characteristics’ frequencies are summarised in table 1 and detailed intervention characteristics can be found in online supplemental appendix D. Interventions were managed and delivered by either local non-governmental organisations (NGOs; n=16)^62–77^ or international NGOs (n=14),^78–91^ or a collaboration between both (n=5).^92–96^ Most interventions were held in Lebanon (n=17), Jordan (n=8), Turkey (n=5) and Iraq (n=3). Of the 38 studies, 13 were held in camps, 12 in urban areas, six in rural areas and four were in mixed settings. Eighteen studies targeted the general population (ie, adult men and women), eight studies targeted women and children, eight studies targeted women only and only one study targeted men only. Twenty-nine studies targeted refugees only, three targeted internally displaced populations and six targeted a mix of refugee/displaced and host populations.

**Table 1:** Frequencies for intervention characteristics of included studies (n=38)

Variable	n		n
*Country*		*Intervention format*	
Lebanon	17	Group (Face-to-Face; F2F)	25
Jordan	8	Individual (F2F)	4
Turkey	5	Online	2
Iraq	3	Mixed (F2F individual and group)	3
Palestine	1	Variable (tailored to individual’s needs)	2
Turkey/Syria	1	Cash-based/Scholarship	2
Lebanon/Jordan	1		
Lebanon/Malaysia	1		
Multi-country (three or more)	1		
*Study design*		*Intervention Provider/Facilitator*	
Cohort	19	Mental health (MH) professional (psychiatrist, psychologist, therapist)	11
Quantitative	8		
Qualitative	7	Mix of MH professionals+HCWs	3
Mixed Methods	4	Healthcare workers (HCWs)	2
Randomised controlled trial	15	Trained non-specialist NGO staff	3
Case study	2	Trained non-specialist peers (refugees or community members)	6
Quasi-experimental	1		
Implementation evaluation	1	Trained non-specialist layperson	5
		Mix of NGO staff+community members	3
		Online application/web-based	2
		No facilitator	2
		Theatre facilitator	1
*Comparator*		*Number of intervention sessions*	
Yes	20	1–4	4
No	18	5–8	8
		9–12	10
		13+	6
		Flexible	7
		Unreported	3
*Displacement status of target population*		*Intervention intensity*	
Refugees	29	Daily	3
Internally displaced people	3	Weekly	20
Mixed (Refugees/Displaced and Host)	6	Monthly	2
		Variable/Flexible (based on participant’s needs)	7
		Unreported	4
*Target population*		*Intervention duration (weeks)*	
All/general population	18	<4	5
Women only	8	4–8	6
Men only	1	>8–12	8
Youth	1	13–19	1
Caregivers only	2	20–25	3
Caregiver(s) and children	8	>20	3
		Unreported	12
*Study Setting*		*IASC MHPSS Intervention Pyramid Layers*	
Camp	13	(1) Basic services and security	1
Urban	12	(2) Community and family support	20
Rural	6	(3) Focused, non-specialised support	7
Mixed (Camp/Urban/and/or Rural)	4	(4) Specialised mental healthcare services	4
Unreported	3	Multi-layered (targeting two or more layers)	6
*Intervention Modality*			
Multimodal/Multidisciplinary (includes two or more of the below types)	10	Narrative exposure therapy	1
Parenting skills	7	Problem solving	1
Community-based participatory intervention	5	Psychoeducation	1
Cognitive behavioural therapy based	3	Behavioural activation	1
Eye Movement Desensitisation and Reprocessing	2	Nature-based (Gardening)	1
Drama therapy	2	Cash-based (Financial)	1
Resilience/Character building	1	Educational Scholarship	1

IASC, Inter-Agency Standing Committee; MHPSS, mental health and psychosocial support; NGO, non-governmental organisation.

#### Intervention modalities, facilitators and IASC MHPSS pyramid layer

MHPSS intervention modalities varied with the majority being multimodal (n=10), that is, including two or more different types, most commonly featuring a combination of stress management, behavioural activation and social support. Other interventions included: parenting skills (n=7),^66–68 70 80 93 94^ community-based participatory (n=5),^71 72 78 81 82^ CBT (n=3),^73 76 91^ EMDR (n=2),^62 97^ drama therapy (n=2), resilience and character building (n=2)^65 74^ and NET (n=1).^96^ Eleven interventions were delivered by mental health specialists, three by a mix of specialists and non-specialists and 20 by trained non-specialists, including healthcare workers, NGO staff, peer refugees and community members. Two interventions were held online and two did not report the facilitator. Eight studies reported integrating facilitator training into their interventions.^76 78 81 83 85 89 95 96^

According to the IASC intervention pyramid for MHPSS in emergency settings, most studies provided layer 2 - community and family support (n=20), while fewer studies offered layer 4 - specialised MHPSS services requiring only experts to deliver such as CBT, NET and EMDR (n=4). Six studies offered a multi-layered approach, targeting three levels of interventions – the individual level, group level (of service users) and the level of the wider community.^63 84 89 92 95 96^

### Feasibility, efficacy-effectiveness, implementation and quality

#### Study design and quality

Intervention designs were mainly single-arm cohort studies (including qualitative, quantitative or mixed methods designs; n=19),^65 66 69–72 75 76 78–85 92 95 96 98^ followed by RCTs (n=15),^62 64 67 68 73 74 77 87 88 90 91 93 94 97 99^ case studies (n=2),^63 86^ quasi-experimental design (n=1)^89^ and implementation evaluation (n=1).^66^

Of the 38 studies, 28 were rated as excellent quality (13 RCTs, 1 quasi-experimental, 13 cohort, 1 implementation evaluation), nine were rated good (2 RCTs, 6 cohort, 1 case study) and one was rated low quality (1 case study). Based on the hierarchy of evidence, RCTs are highest up the hierarchy and thus considered the most evidence-based, followed by quasi-experimental design, cohort and case studies; QA aligned with this hierarchy.

#### Efficacy-effectiveness continuum

Studies were characterised along the RITES efficacy-effectiveness continuum (online supplemental appendix E). Results demonstrated that most studies provided information emphasising *effectiveness* over *efficacy,* scoring ‘4’ or ‘5’ across all domains: (1) Participant characteristics (n=26), (2) Setting (n=32), (3) Flexibility of intervention (n=17) and (4) Clinical relevance (n=21). For instance, most interventions’ participant characteristics were representative of the larger population and settings in which refugee interventions were held. Interventions targeted Arabic-speaking non-clinical adult refugees (men and women) and were held in refugee camps or low socioeconomic neighbourhoods where displaced populations lived.

Some studies, mainly RCTs, scored ‘3’ on the flexibility of interventions (n=13) and clinical relevance domains (n=13). These studies emphasised a balance of both efficacy and effectiveness due to the nature of their study design (ie, RCT), where having a strict protocol, adherence measures and a control group to compare changes in outcomes pre- and post-intervention were deemed necessary.

Studies having a rather strong emphasis on efficacy (scored ‘2’) had specific participant eligibility criteria (eg, clinical diagnosis of mild to moderate depression; n=7), strict protocols and adherence measures (n=4) and a statistically significant reduction in or prevention of a clinical diagnosis (eg, distress and/or PTSD; n=2). No studies scored ‘1’ (strong emphasis on efficacy) in any domain due to interventions being held in humanitarian settings within non-specialised venues, for example, community centres or general healthcare centres as opposed to specialised mental health clinics, targeting non-clinical populations (few studies included participants with clinical diagnoses) and applying flexible or no protocols/adherence measures. Four studies did not report information on the ‘flexibility of intervention’ domain and one study did not report on the ‘clinical relevance’ domain.

#### Ranking across feasibility, implementation and quality

Studies were ranked from highest to lowest according to implementation (ImpRess) and quality (QuADS) scores as these tools were the most comprehensive in assessing the acceptability, effectiveness and quality of interventions (see online supplemental appendices D and F). Feasibility parameters are also reported. Scores ranged from three to seven out of eight feasibility parameters reported, 24–44 out of 52 on implementation and 18–39 out of 39 on quality.

### Sub-analysis of top ten studies

#### Effectiveness parameters

The top ten interventions (online supplemental appendix G) that were most feasible, implementable and high-quality reported on at least three to seven feasibility parameters, had an implementation score range of 39–44 and a quality score range of 34–39.^66 67 73 75 77 81 88 93 94 97^ The study designs included seven RCTs (three of which were pilots),^67 73 77 88 93 94 97^ two cohort community interventions^75 81^ and one implementation evaluation.^66^ Eight interventions were group-based,^66 67 73 75 77 81 88 93 94^ encouraging new social support networks and one was an individual-based EMDR intervention.^97^

Effectiveness parameters were defined as effect size (Cohen’s d), odds ratio and/or p values for quantitative studies and temporal changes to outcomes for qualitative studies. The most common mechanisms of change included enhancing access to social support,^73 75 77 81 88 93^ parenting skills^66 67 73 93 94^ and emotional regulation.^67 73 77 88 94 97^ All ten studies reported that their intervention was effective for primary outcomes, except for the early marriage community intervention, although it did find significant changes for its qualitative outcomes.^81^

#### IC-ADAPT factors analysis

Sub-analysis of the top ten interventions (online supplemental appendix H) demonstrated strong alignment with the IC-ADAPT factors, despite no explicit reference to the framework. The most addressed ADAPT systems were social bonds/networks, roles/identities and safety/security. All interventions aimed to foster social support, trust and community cohesion through group-based delivery, peer facilitation and/or caregiver–child interaction. Seven interventions aimed to restore functional roles, specifically through caregiving education or peer support (eg, CSI, Teaching Recovering Techniques (TRT), MOCEP), rebuilding a sense of agency and competence disrupted by displacement.^67 93 94^ While safety/security was not the main aim, most interventions created a psychologically safe environment by being non-clinical, culturally sensitive and engaging community-based trusted facilitators to deliver the intervention. Justice and existential meaning systems were less addressed, but researchers acknowledged the injustices faced by refugees impacted by war and some recognised the importance of how culture shapes idioms of distress and experiences. Furthermore, culturally sensitive interventions, those that went beyond language and images to organise detailed consultations with cultural representatives to enable cultural relevance throughout the programme, were more acceptable and compatible with participants’ worldviews and beliefs.^67 73 81^

IC principles were well-integrated into interventions; participants reported an enhanced ability to embrace multiple perspectives and identify more choices on how to deal with their challenges, regulate emotions and reconcile difficult experiences. For example, through storytelling or group discussions, participants were encouraged to reflect on their own and others’ feelings and behaviours in a nuanced way.^73 75^ Programmes like gPM+ and TRT fostered cognitive flexibility (flexible problem-solving and self-regulation) under stress^77 88 94^ and integration of trauma narratives without avoidance or overgeneralisation.^73 97^ The combined effects of addressing ADAPT’s psychosocial system disruptions and enhancing IC skills fostered more adaptive functioning in refugees and more favourable outcomes overall. For instance, parenting skills interventions provided caregivers with nuanced response strategies to support child behaviour, leading to enhanced communication, deepened emotional bonds and reduced conflict within the family unit.^94^

## Discussion

This systematic review is the first to evaluate MHPSS interventions targeting non-clinical, Arabic-speaking refugee and displaced adult populations in the MENA region. By applying rigorous quality appraisal tools and analysing intervention outcomes in relation to the IC-ADAPT framework, the review addresses key gaps in the evidence base and provides important insights into culturally relevant public mental health interventions in conflict-affected humanitarian contexts.

A review of 38 studies revealed considerable heterogeneity in intervention design, evaluation methods and implementation approaches. However, key findings and patterns emerged. Most interventions were community-based and group-delivered by non-specialists, targeting families, caregivers or mixed-gender adult populations. Parenting support programmes, multimodal psychosocial interventions and psychotherapeutic modalities dominated the landscape. These approaches align with the IASC’s recommendation to strengthen community and family support (layer 2 of the intervention pyramid), which represented most of the interventions reviewed.^54^

Effectiveness varied across studies, but several demonstrated statistically and clinically significant improvements in outcomes such as parenting practices, emotional distress, PTSD symptoms and child well-being. Ten studies met the highest criteria for feasibility, quality and implementation. Among these, evidence of strong links to IC-ADAPT factors – particularly the ADAPT systems of safety/security, bonds/networks and roles/identities, and IC skills of self-regulation and cognitive flexibility – underscores the framework’s relevance in this context. For example, interventions that enhanced caregiver well-being and reduced harsh parenting (eg, CSI, TRT) implicitly addressed disrupted safety/security and roles/identities, enabling more adaptive responses to chronic stress that also prioritised the family’s well-being.^68 70 80^ The most effective interventions – such as TRT – also explicitly considered social norms, local values, gender roles and constraints related to employment obligations, which increased participant engagement, retention and improved outcomes.^72 74 75 78 81 94^

Findings highlighted a shift from narrowly defined clinical models towards eco-psychosocial approaches. This recognises that non-clinical populations may not necessarily experience distress that meets diagnostic thresholds for mental illness but can still benefit from structured and culturally relevant support through rebuilding psychosocial systems and strengthening skills to encourage more adaptive responses to distress. For example, interventions rooted in community participation, relational healing and cultural relevance showed higher feasibility, acceptability and effectiveness.^72 73 75 82^ Furthermore, designing interventions that are informed by frameworks like IC-ADAPT can facilitate psychosocial healing even in the absence of clinical treatment and support cognitive complexity that fosters community dialogue and integration.

This review is also the first exploration of IC-ADAPT’s relevance to this population. While some work has been done to operationalise ADAPT into an intervention for refugees, both on an individual and group level, these did not fit the geographical region and were excluded.^46 100^

### Implications for future research and practice

This review demonstrates the need for (1) Framework-driven, evidence-based and culturally validated frameworks such as IC-ADAPT from the outset of intervention design, (2) Community-based participatory approaches to co-design and implement interventions, (3) Improving methodological rigour, including larger sample sizes, control/comparison groups and mixed-methods designs, (4) Gender and culturally sensitive interventions that account for caregiving roles, social norms and anti-immigration policies and (5) Expanding beyond symptom reduction to include changes to structural and relational determinants of distress such as enhancing family functioning and cognitive flexibility in line with IC-ADAPT. The review also highlighted that there remains a gap for gender-sensitive interventions targeting men specifically (only one was identified).

### Strengths and limitations

This review’s strengths include its comprehensive scope of study designs, use of multiple validated QA tools and novel application of the IC-ADAPT framework for synthesis. It contributes a critical regional focus to a literature base that has disproportionately examined refugee mental health in high-income, Western host countries.

However, the review also has limitations. Despite broad database and grey literature searches, some relevant interventions may not have been captured, particularly unpublished programmes implemented by local NGOs without formal evaluation. The heterogeneity in intervention design and reporting limited the feasibility of accurate comparisons between quantitative and qualitative outcomes. Furthermore, while the IC-ADAPT analysis yielded valuable insights, it relied on author interpretation due to the absence of direct reporting on these frameworks in the reviewed studies.

The QA tools selected were shown to be the most valid and reliable in their field; however, not all criteria were relevant and some required modification, redefinition or other adaptation for humanitarian settings. As such, two authors (LB and EB) developed a thorough scoring guide to re-interpret most criteria to ensure scoring alignment and relevance to setting, study design and context. A gap in the literature was identified for QA tools relevant to humanitarian settings and an opinion piece is being drafted by the authors for publication.

## Conclusion

This review highlights the value of culturally sensitive, non-clinical MHPSS interventions that are feasible, effective and implementable in humanitarian settings for Arabic-speaking refugee populations in MENA. It supports using IC-ADAPT as a guiding framework for developing and evaluating culturally grounded MHPSS interventions. Our findings underscore the importance of addressing structural and relational determinants of distress and offer practical guidance for scaling interventions through non-specialist delivery. These results can also be generalised to non-refugee, underserved populations. Populations experiencing chronic stress can benefit from MHPSS frameworks like IC-ADAPT, which are transdiagnostic, adaptable, recognise idioms of distress and can be applied to multiple settings (eg, healthcare systems, schools and prisons). Findings can inform policy efforts to embed MHPSS into broader community and humanitarian systems in the region.

## Supplementary material

10.1136/bmjgh-2025-021128 online supplemental appendix 1

## Data Availability

All data relevant to the study are included in the article or uploaded as supplementary information.

## References

[1] European Commission (2024). Forced displacement: refugees, asylum-seekers, and internally displaced persons (IDPS). https://civil-protection-humanitarian-aid.ec.europa.eu/what/humanitarian-aid/forced-displacement_en#:~:text=As%20of%20May%202024%2C%20the,global%20figures%20for%20forced%20displacement.

[2] UNHCR (2024). Syria refugee crisis. https://www.unrefugees.org/emergencies/syria/.

[3] UNHCR (2022). Refugee data finder: key indicators. https://www.unhcr.org/refugee-statistics/.

[4] UNHCFR (2024). UNHCR lebanon at a glance. https://www.unhcr.org/lb/at-a-glance#:~:text=Lebanon%20remains%20the%20country%20hosting,11%2C645%20refugees%20of%20other%20nationalitiesu.

[5] UNHCfR (2024). UNHCR country report: Jordan. https://www.unhcr.org/uk/countries/jordan.

[6] ACAPS (2024). Country analysis: Jordan. https://www.acaps.org/en/countries/jordan.

[7] UNRaWA (2024). Palestine refugees. https://www.unrwa.org/palestine-refugees.

[8] Kazour F, Zahreddine NR, Maragel MG (2017). Post-traumatic stress disorder in a sample of Syrian refugees in Lebanon. Compr Psychiatry.

[9] Kerbage H, Marranconi F, Chamoun Y (2020). Mental Health Services for Syrian Refugees in Lebanon: Perceptions and Experiences of Professionals and Refugees. Qual Health Res.

[10] Grasser LR (2022). Addressing Mental Health Concerns in Refugees and Displaced Populations: Is Enough Being Done?. Risk Manag Healthc Policy.

[11] Boyd-MacMillan EM, DeMarinis V (2020). 'Section four, mental health, psychosocial support and social and emotional learning’,. In the learning passport: research and recommendations report.

[12] Boyd-MacMillan EM, DeMarinis V (2020). Learning passport: curriculum framework (IC-ADAPT SEL high level programme design).

[13] World Health Organization (2019). Mental health in emergencies.

[14] McGregor LS, Melvin GA, Newman LK (2016). An exploration of the adaptation and development after persecution and trauma (ADAPT) model with resettled refugee adolescents in Australia: A qualitative study. Transcult Psychiatry.

[15] Yohani S (2015). Applying the ADAPT Psychosocial Model to War-Affected Children and Adolescents. Sage Open.

[16] Hassan G, Kirmayer L, Mekki-Berrada A (2025). Culture, context and the mental health and psychosocial wellbeing of Syrians: a review for mental health and psychosocial support staff working with Syrians affected by armed conflict.

[17] Rana M, Boyd‐MacMillan E (2024). Coloniality, violence, and intergenerational trauma among displaced Syrians: An interdisciplinary scoping review. Mental Health Science.

[18] Atallah DG, Shapiro ER, Al-Azraq N (2018). Decolonizing qualitative research through transformative community engagement: critical investigation of resilience with Palestinian refugees in the West Bank. Qual Res Psychol.

[19] Bracken P, Fernando S, Alsaraf S (2021). Decolonising the medical curriculum: psychiatry faces particular challenges. Anthropol Med.

[20] Kohrt BA, Rasmussen A, Kaiser BN (2014). Cultural concepts of distress and psychiatric disorders: literature review and research recommendations for global mental health epidemiology. Int J Epidemiol.

[21] Jordan Times (2025). UNHCR says “significant” number of Syrian refugees return home from Jordan. https://www.jordantimes.com/news/local/unhcr-says-significant-number-syrian-refugees-return-home-jordan.

[22] McKenna DF (2024). Sally. Syrian refugee still fearful after regime collapse. https://www.bbc.co.uk/news/articles/cpvn1zjdyejo.

[23] Marks J (2024). Ensuring a safe, secure, and dignified future for displaced Syrians. https://www.refugeesinternational.org/reports-briefs/ensuring-a-safe-secure-and-dignified-future-for-displaced-syrians/.

[24] Silove D (2022). Visions from the past: Reflecting on the history of epidemiological research in the refugee and post-conflict mental health field. Torture.

[25] Silove D (2006). Handbook of international disaster psychology: refugee mental health, vol 3.

[26] Silove D (2000). Psychological debriefing: theory, practice and evidence.

[27] Silove D (1999). The psychosocial effects of torture, mass human rights violations, and refugee trauma: toward an integrated conceptual framework. J Nerv Ment Dis.

[28] Boyd-MacMillan E, Fearon P, Ptolomey A (2016). I SEE! Scotland: Tackling Sectarianism and Promoting Community Psychosocial Health. *JSS*.

[29] Boyd-MacMillan E, University of Cambridge (2016). Increasing Cognitive Complexity and Collaboration Across Communities: Being Muslim Being Scottish. *JSS*.

[30] Brodbeck FC, Kugler KG, Fischer JA (2021). Group-level integrative complexity: Enhancing differentiation and integration in group decision-making. Group Process Intergr Relat.

[31] Park G, DeShon RP (2018). Effects of group-discussion integrative complexity on intergroup relations in a social dilemma. Organ Behav Hum Decis Process.

[32] Tetlock PE, Skitka L, Boettger R (1989). Social and cognitive strategies for coping with accountability: conformity, complexity, and bolstering. J Pers Soc Psychol.

[33] Boyd-MacMillan E, Campbell C, Furey A (2016). An IC Intervention for Post-Conflict Northern Ireland Secondary Schools. *JSS*.

[34] Fearon P, Boyd-MacMillan E, University of California - Berkeley (2016). Complexity Under Stress: Integrative Approaches to Overdetermined Vulnerabilities. JSS.

[35] Woodard SR, Chan L, Conway LG (2021). In Search of the Cognitively Complex Person: Is There a Meaningful Trait Component of Cognitive Complexity?. Pers Soc Psychol Rev.

[36] Silove D (2013). The ADAPT model: a conceptual framework for mental health and psychosocial programming in post conflict settings. Intervention.

[37] Wells R, Abo-Hilal M, Steel Z (2020). Community readiness in the Syrian refugee community in Jordan: A rapid ecological assessment tool to build psychosocial service capacity. Am J Orthopsychiatry.

[38] Boyd-MacMillan EM, DeMarinis V (2021). Unpublished responses to Swedish partner questions regarding why it is valuable to raise IC (what difference does it make). [Unpublished work].

[39] Tuckman BW (1965). Integrative complexity and attitudinal orientation. Percept Mot Skills.

[40] Suedfeld P, Tetlock PE (2014). Integrative Complexity at Forty: Steps Toward Resolving the Scoring Dilemma. Polit Psychol.

[41] Frauenfelder KJ (1974). Integrative Complexity and Extreme Responses. Psychol Rep.

[42] Suedfeld P, Coren S (1992). Cognitive correlates of conceptual complexity. Pers Individ Dif.

[43] Tetlock PE (1984). Cognitive style and political belief systems in the British House of Commons. J Pers Soc Psychol.

[44] Wells R, Lawsin C, Hunt C (2018). An ecological model of adaptation to displacement: individual, cultural and community factors affecting psychosocial adjustment among Syrian refugees in Jordan. *Glob Ment Health (Camb*).

[45] Tay AK, Silove D (2017). The ADAPT model: bridging the gap between psychosocial and individual responses to mass violence and refugee trauma. Epidemiol Psychiatr Sci.

[46] Tay AK, Miah MAA, Khan S (2019). Theoretical background, first stage development and adaptation of a novel Integrative Adapt Therapy (IAT) for refugees. Epidemiol Psychiatr Sci.

[47] Zoellner LA, Bentley JA, Feeny NC (2021). Reaching the Unreached: Bridging Islam and Science to Treat the Mental Wounds of War. Front Psychiatry.

[48] United Nations High Commissioner for Refugees (UNHCR) (2024). Data and statistics: global trends [online global trends report]. https://www.unhcr.org/global-trends-report-2023.

[49] Dalgaard NT, Thøgersen MH, Riber K, Rousseau C, De Haene L (2020). Working with refugee families: trauma and exile in family relationships.

[50] Wells R, Steel Z, Abo-Hilal M (2016). Psychosocial concerns reported by Syrian refugees living in Jordan: systematic review of unpublished needs assessments. *Br J Psychiatry*.

[51] Summerfield D (1999). A critique of seven assumptions behind psychological trauma programmes in war-affected areas. Soc Sci Med.

[52] Wells DR, Wells R, Lawsin C (2015). Understanding psychological responses to trauma among refugees: the importance of measurement validity in cross-cultural settings. J Proc R Soc N S W.

[53] Kamali M, Munyuzangabo M, Siddiqui FJ (2020). Delivering mental health and psychosocial support interventions to women and children in conflict settings: a systematic review. BMJ Glob Health.

[54] Committee I-AS (2021). The common monitoring and evaluation framework for mental health and psychosocial support in emergency settings: with means of verification (version 2.0).

[55] Page MJ, McKenzie JE, Bossuyt PM (2021). The PRISMA 2020 statement: an updated guideline for reporting systematic reviews. BMJ.

[56] Eldridge SM, Lancaster GA, Campbell MJ (2016). Defining Feasibility and Pilot Studies in Preparation for Randomised Controlled Trials: Development of a Conceptual Framework. PLoS ONE.

[57] Wieland LS, Berman BM, Altman DG (2017). Rating of Included Trials on the Efficacy–Effectiveness Spectrum: development of a new tool for systematic reviews. J Clin Epidemiol.

[58] Streater A, Spector A, Aguirre E (2016). ImpRess: an Implementation Readiness checklist developed using a systematic review of randomised controlled trials assessing cognitive stimulation for dementia. BMC Med Res Methodol.

[59] Harrison R, Jones B, Gardner P (2021). Quality assessment with diverse studies (QuADS): an appraisal tool for methodological and reporting quality in systematic reviews of mixed- or multi-method studies. BMC Health Serv Res.

[60] Popay J, Roberts H, Aj S (2005). Guidance on the conduct of narrative synthesis in systematic reviews final report. J Epidemiol Community Health.

[61] De Brún C (2013). Finding the evidence.

[62] Yurtsever A, Konuk E, Akyüz T (2018). An Eye Movement Desensitization and Reprocessing (EMDR) Group Intervention for Syrian Refugees With Post-traumatic Stress Symptoms: Results of a Randomized Controlled Trial. Front Psychol.

[63] Womersley G, Arikut-Treece Y (2019). Collective trauma among displaced populations in Northern Iraq: A case study evaluating the therapeutic interventions of the Free Yezidi Foundation. Interv J Ment Health Psychosoc Support Confl Affect Areas.

[64] Weinstein N, Khabbaz F, Legate N (2016). Enhancing need satisfaction to reduce psychological distress in Syrian refugees. J Consult Clin Psychol.

[65] Sakhi S, Kreidie LH, Wardani F (2022). Drama therapy as a mental health intervention for women in the shatila refugee camp, Lebanon. Intervention.

[66] Ponguta LA, Issa G, Aoudeh L (2019). Implementation Evaluation of the Mother‐Child Education Program Among Refugee and Other Vulnerable Communities in Lebanon. New Dir Child Adolesc Dev.

[67] Leckman JF (2020). Effects of the Mother-Child Education Program on Parenting Stress and Disciplinary Practices Among Refugee and Other Marginalized Communities in Lebanon: A Pilot Randomized Controlled Trial. J Am Acad Child Adolesc Psychiatry.

[68] Miller KE, Koppenol-Gonzalez GV, Arnous M (2020). Supporting Syrian families displaced by armed conflict: A pilot randomized controlled trial of the Caregiver Support Intervention. Child Abuse Negl.

[69] Lancaster SL, Gaede C (2020). A test of a resilience based intervention for mental health problems in Iraqi internally displaced person camps. Anxiety Stress Coping.

[70] Lakkis NA, Osman MH, Aoude LC (2020). A Pilot Intervention to Promote Positive Parenting in Refugees from Syria in Lebanon and Jordan. Front Psychiatry.

[71] Jirmanus LZ, Ziadee M, Usta J (2021). Confronting Structural Inequities: The Limits of Participation when Developing a Community Health Intervention with Syrian Refugees and Host Communities in Lebanon. Soc Sci Med.

[72] James LE, Welton-Mitchell C, Michael S (2021). Development and Testing of a Community-Based Intervention to Address Intimate Partner Violence among Rohingya and Syrian Refugees: A Social Norms-Based Mental Health-Integrated Approach. Int J Environ Res Public Health.

[73] Eskici HS, Hinton DE, Jalal B (2023). Culturally adapted cognitive behavioral therapy for Syrian refugee women in Turkey: A randomized controlled trial. Psychol Trauma.

[74] Jalal KD (2022). The effects of psychodrama intervention on intimate partner violence and quality of life: trial of Syrian refugee abused women.

[75] Bunn M, Marsh J, Haidar A (2022). Sharing Stories Eases Pain: Core Relational Processes of a Group Intervention with Syrian Refugees in Jordan. J Spec Group Work.

[76] Meredith AB, Elizabeth AY, Salah A (2022). Sustaining psychotherapist effectiveness and independence: An exploratory study with displaced persons in Kurdistan, Iraq. Traumatology (Tallahass Fla).

[77] Acarturk C, Uygun E, Ilkkursun Z (2022). Group problem management plus (PM+) to decrease psychological distress among Syrian refugees in Turkey: a pilot randomised controlled trial. BMC Psychiatry.

[78] Veale A, Shanahan F, Hijazi A (2020). Engaging men to promote resilient communities among Syrian refugees in Lebanon. *Intervention*.

[79] Talhouk SN, Chalak A, Kamareddine Z (2021). Vertical gardening and Syrian women refugees in Lebanon: an exploratory study on motivation for gardening and depression relief. Local Environ.

[80] Sim AL, Bowes L, Maignant S (2021). Acceptability and Preliminary Outcomes of a Parenting Intervention for Syrian Refugees. Res Soc Work Pract.

[81] Sieverding M, Bteddini D, Mourtada R (2022). Design and Implementation of the Amenah Early Marriage Pilot Intervention Among Syrian Refugees in Lebanon. Glob Health Sci Pract.

[82] Sahyoun NR, Jamaluddine Z, Choufani J (2019). A mixed-methods evaluation of community-based healthy kitchens as social enterprises for refugee women. BMC Public Health.

[83] Powell TM, Qushua N (2023). A qualitative study of a mental health awareness intervention for Jordanian and resettled Syrian refugees in host communities. Int J Soc Psychiatry.

[84] Lilleston P, Winograd L, Ahmed S (2018). Evaluation of a mobile approach to gender-based violence service delivery among Syrian refugees in Lebanon. Health Policy Plan.

[85] Khoury B, Daouk S (2022). Problem-Solving Skills Groups for Female Syrian Refugees in Lebanon: A Study of a Mental Health Intervention. J Refug Stud.

[86] Hagen‐Zanker J, Ulrichs M, Holmes R (2018). What are the effects of cash transfers for refugees in the context of protracted displacement? Findings from Jordan. *Int Social Security Review*.

[87] Cuijpers P, Heim E, Abi Ramia J (2022). Effects of a WHO-guided digital health intervention for depression in Syrian refugees in Lebanon: A randomized controlled trial. PLoS Med.

[88] Bryant RA, Bawaneh A, Awwad M (2022). Effectiveness of a brief group behavioral intervention for common mental disorders in Syrian refugees in Jordan: A randomized controlled trial. PLoS Med.

[89] Bruno W, Kitamura A, Najjar S (2019). Assessment of mental health and psycho-social support pilot program’s effect on intended stigmatizing behavior at the Saftawi Health Center, Gaza: a cross-sectional study. J Ment Health.

[90] Akhtar A, Malik A, Ghatasheh M (2021). Feasibility trial of a brief scalable psychological intervention for Syrian refugee adolescents in Jordan. Eur J Psychotraumatol.

[91] Acarturk C, Uygun E, Ilkkursun Z (2022). Effectiveness of a WHO self-help psychological intervention for preventing mental disorders among Syrian refugees in Turkey: a randomized controlled trial. World Psychiatry.

[92] Yassin N, Taha AA, Ghantous Z (2018). Evaluating a Mental Health Program for Palestinian Refugees in Lebanon. J Immigrant Minority Health.

[93] Miller KE, Chen A, Koppenol‐Gonzalez GV (2023). Supporting parenting among Syrian refugees in Lebanon: a randomized controlled trial of the caregiver support intervention. Child Psychology Psychiatry.

[94] El-Khani A, Cartwright K, Maalouf W (2021). Enhancing Teaching Recovery Techniques (TRT) with Parenting Skills: RCT of TRT + Parenting with Trauma-Affected Syrian Refugees in Lebanon Utilising Remote Training with Implications for Insecure Contexts and COVID-19. Int J Environ Res Public Health.

[95] Budosan B, Benner MT, Abras B (2016). Evaluation of one mental health/psychosocial intervention for Syrian refugees in Turkey. International NGO Journal.

[96] Viller Hansen AK, Sloth Hansen-Nord N, Smeir I (2017). Impact of NET on torture survivors in the MENA region. Torture.

[97] Acarturk C, Konuk E, Cetinkaya M (2015). EMDR for Syrian refugees with posttraumatic stress disorder symptoms: results of a pilot randomized controlled trial. Eur J Psychotraumatol.

[98] Al-Rousan T, Fredricks K, Chaudhury S (2018). Improving peace and well-being among Syrian refugee youth through a higher education initiative in Jordan. Med Confl Surviv.

[99] Ruggeri K, Jarke H, El-Zein L (2021). Mental health and decisions under risk among refugees and the public in Lebanon. *Humanit Soc Sci Commun*.

[100] Tay AK, Mung HK, Miah MAA (2020). An Integrative Adapt Therapy for common mental health symptoms and adaptive stress amongst Rohingya, Chin, and Kachin refugees living in Malaysia: A randomized controlled trial. PLoS Med.

