# Supplementary material for: A systematic review of MHPSS interventions targeting non-clinical Arabic-speaking refugees and/or displaced populations in the MENA region
Source: BMJ Glob Health. 2026 Jul 30;11(7):e021128. doi: 10.1136/bmjgh-2025-021128 (PMC13423163; doi:10.1136/bmjgh-2025-021128)
Supplement: online supplemental appendix 1 [file bmjgh-11-7-s001.docx]

APPENDICES

[Appendix A: MEDLINE Search Strategy for MHPSS interventions targeting MENA adult refugees in the MENA region 2](#_Toc230086691)

[Appendix B: Quality Assessment (QA) Tools, Definitions, Parameters, and Rationale for Selection 4](#_Toc230086692)

[Appendix C: National Health Services (NHS) Hierarchy of Evidence Pyramid 7](#_Toc230086693)

[Appendix D. Study Characteristics of Interventions Ranked from Highest to Lowest for Feasibility, Implementation, and Quality (n= 38) 8](#_Toc230086694)

[Appendix E: Visual Presentation of the Efficacy-Effectiveness of Included Studies (n= 38) 39](#_Toc230086695)

[Appendix F: Scatterplot showing Quality Rating and Most-to-Least Feasible and Implementable Studies (n= 38). 40](#_Toc230086696)

[Appendix G: IC-ADAPT Links for top ten studies 41](#_Toc230086697)

[Appendix H: IC-ADAPT synthesis of top ten studies 53](#_Toc230086698)

# Appendix A: MEDLINE Search Strategy for MHPSS interventions targeting MENA adult refugees in the MENA region

1. IC-ADAPT Framework

1 (Systems based or community program* or "IC adapt" or ICAdapt or Adapt model or "adaptation and development after persecution and trauma" or "integra* complexity" or resilien* or empathy or emotion* regulation* or emotion* selfregulation* or emotion* self regulation* or implicit attitude* or negotiat*or group dynamic* or family group* or wellbeing or well being or mental health or mental* ill* or mental disorder* or zero sum mindset*).ti,ab. 421539

2 Resilience, psychological/ or empathy/ or emotional regulation/ or attitude/ or negotiating/ or group processes/ or exp mental health/ or exp mental disorders/ 1522417

3 "freedom of movement".ti,ab. or exp "Freedom of Movement"/ 726

4 (Bond* or network* or ritual* or ceremon* or grief* or griev* or mourn* or attachment* or loss or Social or society or family or friend* or work colleague or profession* or status or religion or communit* or tribe* or tribal* or sect or elders or chieftain*).ti,ab. 5156214

5 Interpersonal relations/ or social networking/ or ceremonial behavior/ or grief/ or object attachment/ or social support/ or friends/ or exp family/ or social structure/ or social status/ or religion/ or community networks/ 510835

6 (survival response or reliance or reliant or dependen* or adaptive response* or maladaptive response* or normative defensive response* or emotional safety or psychological safety or physical safety or emotional security or psychological security or physical security or persecution).ti,ab. 1871017

7 Adaptation, psychological/ or safety/ or conflict, psychological/ 159530

8 (justice or injustice or human right* or trust* or distrust* or mistrust or resent* or grievance* or restorative or humiliat* or degrad* or dehuman* or infrahuman* or infra human* or dignity or indignity or moral injury).ti,ab. 582662

9 Social justice/ or human rights/ or trust/ or respect/ or shame/ 43516

10 (cultural norm* or social norm* or societal norm* or normative influence* or cultural adaptation or cultural characteristic* or values or roles or identit* or acculturation or assimilat* or bicultural or anomie or withdraw* or depersonal* or dereal*).ti,ab. 2013553

11 Culture/ or social norms/ or cultural characteristics/ or social values/ or role/ or social identification/ or acculturation/ or anomie/ or depersonalization/ 100809

12 (existential or belief* or believ* or religion or faith or spiritual* or alienat* or fragment* or differentiation or integrat*).ti,ab. 1872482

13 Existentialism/ or culture/ or religion/ or spirituality/ or social alienation/ or social integration/ 59076

14 (conflict resolution or negotiat* or group think or group process* or intergroup competition* or inter group competition* or intergroup conflict* or inter group conflict* or peaceful engagement or inter group conflict* or intergroup conflict* or ingroup or outgroup or "in group" or "out group" or "expression of values" or awareness or flexib* or "stressor enhancing mindset" or flexible mindset or attitude* or opinion* or polari*).ti,ab. 1004345

15 Negotiating/ or group processes/ or awareness/ or attitude/ 94777

16 or/1-15 11291238

1. Population of interest

17 (refugee* or (displaced adj2 (person* or people* or population*)) or asylum seeker* or mobile population* or internally displaced or involuntary mobility or voluntary mobility or immobile population* or mobile population*).ti,ab. or refugees/ 18125

18 (Assyrian or sunni or shia or shiaa or "shia'a" or shiite* or Yazidi* or Armenian or Armenia or "Church of the East " or druz* or allawite or alawite or alawi or kurds or Kurdish or arab or arabs or orthodox christian* or Chaldean Catholic* or Coptic Orthodox or Greek Catholic* or Greek Orthodox or Roman Catholic* or Maronite* or Protestant* or Syriac or Bedouin* or Zaatari or Azraq or jew or jews or jewish or muslim* or islam* or ismailis or "isma’ilis" or Bahai or "Baha’i" or Kakai or "Kaka’i" or Turkmen or Zorastrian* or Safardic).ti,ab. 37092

19 (Lebanese or Syrian* or Israeli* or Turkish or turks or Egyptian* or Palestinian* or Bahraini* or Iraqi* or Jordanian or Kuwaiti or omani or Qatari or arabs or emirati or Yemeni or Levantine* or Kurdish).ti,ab. 71706

1. Geography

20 (Lebanon or Syria or Israel or turkey or Egypt or palestine or bahrain or Iraq or Jordan or Kuwait or oman or Qatar or Saudi arabia or uae or united arab emirates or yemen or Arabian gulf or Arabian peninsula or persian gulf or persian peninsula or levant or middle east*).ti,ab. 152604

21 exp egypt/ or middle east/ or exp bahrain/ or exp iraq/ or exp israel/ or exp jordan/ or exp kuwait/ or exp lebanon/ or exp oman/ or exp qatar/ or exp saudi arabia/ or exp syria/ or exp turkey/ or exp united arab emirates/ or exp yemen/ 138264

22 (Cairo or Istanbul or Baghdad or Riyadh or Ankara or dubai or sharjah or ajman or Alexandria or Jeddah or amman or izmir or tel aviv or mosul or mashhad or Isfahan or Damascus or abu dhabi or basra or doha or Kuwait city or Beirut or Sanaa or irbid or gaza city or mecca or Aleppo or erbil or muscat or najaf).ti,ab. 32808

23 or/18-22 271718

IV. Intervention

24 (Non-intervention* or model* or theor* or pre-assess* or post-assess* or ("pre- and post*" adj assess*) or "follow* up" or followup or control* or rct or trial* or intervention*).ti,ab,kf,kw. or exp crisis intervention/ or exp psychosocial intervention/ or exp models, theoretical/ or exp follow-up studies/ or exp cohort studies/ 11352380

25 16 and 17 and 23 and 24 973

26 16 and 17 and 23 2118

27 26 not 25 1145

# Appendix B: Quality Assessment (QA) Tools, Definitions, Parameters, and Rationale for Selection

| **QA Tool** | **Definition** | **Parameters and Scoring** | **Rationale for Tool Selection** |
| --- | --- | --- | --- |
| **Feasibility and acceptability**  **(Eldridge et al., 2016)** | *Feasibility* of an intervention looks at whether it is possible to run an intervention in this specific population in challenging circumstances.(71, 75) | 1. Standard deviation of outcome measure 2. Participant willingness to be randomised 3. Willingness of clinicians to recruit participants 4. Number of eligible participants 5. Characteristics of proposed outcome measure 6. Follow-up & response rates 7. Availability of data needed or usefulness and limitations of a particular database 8. Time needed to collect/analyse data.   No scoring guidelines were provided by the tool authors, so the two authors (LB & EB) conducting the scoring developed a scoring guide, where parameters were assessed and categorised as follows: applicable and reported, not applicable, or applicable but unreported. Studies that had a greater number of applicable and reported parameters were more likely to be feasible than those with applicable but unreported parameters. | The parameters from Eldridge et al. were initially identified for randomised controlled trials, and they remain the most comprehensive assessment guidelines for feasibility and acceptability of complex interventions, in line with the UK Medical Research Council (MRC). |
| **Efficacy and effectiveness**  **(RITES, 2017)** | “*Efficacy* can be defined as the performance of an intervention under ideal and controlled circumstances, whereas *effectiveness* refers to its performance under ‘real-world' conditions.” (76)  Efficacy measures whether the intervention produces the expected result, whereas effectiveness measures the degree of beneficial effect under ‘real world’ clinical settings.(77, 78)  Efficacy trials will have high internal validity, while effectiveness trials will have high external validity. Thus, distinguishing between both becomes very important in evaluating interventions as their internal and external validity are interrelated to efficacy and effectiveness, respectively.(79) | 1. Participant characteristics 2. Trial setting 3. Flexibility of intervention 4. Clinical relevance of experimental and comparison intervention(s)   Based on a five-point Likert scale, the evidence deriving from each of the four domains was rated: 1 = strong emphasis on efficacy; 2 = rather strong emphasis on efficacy; 3 = balanced emphasis on both efficacy and effectiveness; 4 = rather strong emphasis on effectiveness; 5 = strong emphasis on effectiveness; NA = information not available.  Although RITES scores are not used to evaluate the quality of studies, they are used to determine where studies fall on the efficacy-effectiveness spectrum for each domain. Higher scores reflect high effectiveness, and lower scores reflect high efficacy. In this systematic review, total scores were not considered as they did not demonstrate relevance to the efficacy/effectiveness spectrum. | RITES is the “first tool systematically designed specifically for characterizing evidence from completed trials along an efficacy-effectiveness  continuum for retrospective use in systematic  reviews.” It was selected as it provides a flexible framework to assess interventions across this continuum by evaluating four parameters.(72) |
| **Implementation**  **(ImpRess, 2016)** | Intervention implementation is important to evaluate as it demonstrates the applicability of an intervention in real life settings.  According to the UK MRC’s guidance for evaluating complex interventions, interventions may have limited effects due to various reasons (e.g., weaknesses in its design or not properly being implemented). It may also be implemented differently, yet still attain favourable outcomes. As such, the MRC defines implementation as the ability to capture fidelity (intervention being delivered as intended) and dose (quantity of intervention implemented). (80) | The ImpRess checklist (Streater et al., 2016) was applied to assess implementation of interventions. Parameters according to the ImpRess checklist are framed around ten themes: Motivation, theory of change, implementation context, experience, planning consultations, delivery collaborations, manager support, employee support, resources, and population characteristics (73)  The ImpRess checklist has 26 criteria, where scoring is determined on a 3-point scale as follows: 0 = no information provided, 1= partially answered, 2= question is fully answered. There is no weighting given to a particular theme and the overall score is used to determine implementation readiness. Total scores range from 0 to 52, with higher scores indicating greater likelihood of implementation. | The ImpRess checklist was originally developed to assess the intervention implementation of a study on cognitive stimulation for dementia. It aligns with the operational definition and assessment of intervention implementation as described by the MRC Framework for Complex Interventions. The framework represents one of the more comprehensive guidance for assessing MHPSS intervention implementation. |
| **Risk of bias (quality) assessment**  **(QuADS, 2021)** | Assessing the quality of studies requires researchers to critically evaluate the degree to which researchers running the study have taken the necessary steps to ensure the highest validity and reliability, relevance to the research question(s), and lowest bias in their findings.(81) | The QuADS tool (Harrison et al., 2021) was designed specifically to appraise qualitative, mixed, and multimethod studies in health services research. QuADS was originally developed for psychological research but later adapted after demonstrating wider relevance and application to health services.  The tool has 13 criteria and allows for scoring on a 4-point Likert scale of 0 to 3: 0 = no information, 1 = little information, 2 = some information, 3 = detailed information. Criterion includes assessing bias, standard and availability of evidence-based tools and the evidence of quality of reporting with a variety of study designs. Total scores range from 0 to 39, with higher scores indicating lower risk of bias and higher quality levels. Total scores are converted into percentages and quality rating is determined as follows: excellent – more than 80%; good – between 50% and 80%; low – under 50%. | While there are many popular risk of bias tools, most are specific to one type of study design. QuADS is an evidence-based alternative to other tools (e.g., GRADE, Cochrane RoB 2 for RCTs only) and is one of the few tools that can be applied to a wide range of study designs. |

# Appendix C: National Health Services (NHS) Hierarchy of Evidence Pyramid


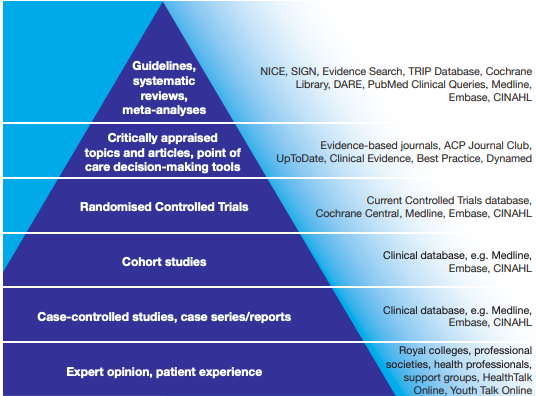


# Appendix D. Study Characteristics of Interventions Ranked from Highest to Lowest for Feasibility, Implementation, and Quality (n= 38)

| **Study** | **Country** | **Population** | **Interven-tion**  **(# sessions – IASC level)** | **Comparison group** | **Assessed for eligibility** | ***N*** | **Recruitment Methods** | **Outcomes +**  **Measures** | **Results** | **Follow-**  **up**  **(mo.)** | **Feasibility** | **ImpRess (0-52)** | **QuADS (0-39)** |
| --- | --- | --- | --- | --- | --- | --- | --- | --- | --- | --- | --- | --- | --- |
| [Miller et al](https://acamh.onlinelibrary.wiley.com/doi/pdfdirect/10.1111/jcpp.13668), 2023 ^62^ | Lebanon | Syrian refugee caregivers | Group Parenting skills  (9 – II) | WLC | 480 | 476 | *Purposive Sampling*  Collaboration with local community-based organizations (CBOs) in two areas of Tripoli that are home to large Syrian refugee communities. Recruitment entailed community breakfasts, door-to-door recruitment, outreach visits to settings where men commonly gather, and word of mouth. | Parental skills (DPS), parenting knowledge (K-10), harsh parenting (WEMWBS), caregiver stress, stress management | No significant change on overall parenting skills at endline or a significant effect on overall parenting skills among participants receiving the full intervention. The CSI showed beneficial effects in the full sample at endline and follow-up on harsh parenting (d=.17,p<.05;d=.19,p<.05), parenting knowledge (d=.63,p<.001;d=.50,p<.001), and caregiver distress (d=.33,p<.001;d=.23,p<.01). No effects found on parental warmth and responsiveness, psychosocial wellbeing, stress, or stress management. Changes in caregiver wellbeing partially mediated the impact of the CSI on harsh parenting, accounting for 37% of the reduction in harsh parenting | 3 | 7 app, 0 non-app, 1 no info | 44 | 38 |
| [El-Khani et al](https://www.proquest.com/docview/2565252766?pq-origsite=gscholar&fromopenview=true), 2021 ^63^ | Lebanon | Displaced Syrian families with children | TRT+P  (2 parent sessions, II) | WLC | 565 | 119 | *Purposive Sampling*  Teachers gave children aged 9–12 years attending three schools where local NGO ran activities a study participant information sheet to take home to their caregivers with an option to ‘opt out’ of the study. No caregivers returned the sheet or showed interest to opt out. | Childhood anxiety-related disorder (CRIES-1), DSRS, SCARE, Parenting (PS), PTSD symptoms (IES-R), Stress-Anxiety-depression (DASS) | Caregivers in the TRT + P group reported significant reductions in depression, anxiety, and stress. | 3 | 7 app, 0 non app, 1 no info | 42 | 37 |
| [Eskici et al](https://pubmed.ncbi.nlm.nih.gov/34618479/), 2021 ^64^ | Turkey | Syrian refugee women | Group  CA-CBT  (7, IV) | TAU | 89 | 23 | *Purposive Sampling*  NGOs that provide psychosocial support to Syrian women were contacted by local NGO facilitator RASASA to collaborate and refer potential participants. The research team (Arabic-speaking university students with training in similar studies) conducted face-to-face interviews with potential participants to brief them on the study. | Feasibility (Dropout rates, adverse events), Anxious-depressive distress (HSCL-25), PTSD symptoms (HTQ) | CA-CBT had a large effect on PTSD (HTQ d=1.17) and nearly medium effect sizes for anxious–depressive distress (HSCL d= .40). There were also low drop-out rates and an absence of adverse events. | 0 | 7 app, 0 non-app, 1 no info | 44 | 35 |
| [Ponguta et al](https://www.sciencedirect.com/science/article/pii/S0890856720300666), 2020 ^65^ | Lebanon | Mother-child dyads of Palestinian and Syrian refugees, and low SES Lebanese families | MOCEP  (25, II) | WLC | 147 | 106 | *Convenience Sampling*  Recruitment was conducted in partnership with community centres, and included sending letters, holding community gatherings and town hall meetings, and following up as needed via telephone calls. | Disciplinary style, Maternal knowledge and practices, Mother’s levels of parenting stress, perceptions of social interaction and subjective support, perceptions of distress, quality of life, Mother’s assessment of husband's involvement in interaction with the mother herself, child, and people in community, maternal empowerment  (DSQ, BPP, SDQ, PSI-S, DSSI-SF, IDS, WHO-5, FIQ, WEQ, Bear/Dragon task, Shape Stroop, ALEF, BSRA, PPSS,  Dyadic interactions) | Forty families (38%) withdrew early from the study. After completing the program, mothers in the intervention group showed a reduction in their harsh parenting practices, as indexed by the Disciplinary Style Questionnaire (Cohen’s d= 0.76, 95% CI= 1.24,0.27) and in their level of parenting stress, as indexed by the Parenting Stress Index Short Form (PSI-SF; Cohen’s d= 0.90, 95% CI=1.39,0.40). Exploratory but underpowered analyses of dyadic interactions revealed reductions in the PSI were associated with a reduction in harsh parenting after the intervention. However, we did not detect any positive impact on behavioural or emotional outcomes among the children | 0 | 7 app, 0 non-app, 1 no info | 40 | 38 |
| [Acarturk et al, 2015](https://www.tandfonline.com/doi/pdf/10.3402/ejpt.v6.27414) ^66^ | Turkey Syrian border | Syrian refugees | Pilot EMDR  (7, IV) | WLC | 45 | 29 | *Random sampling*  820 adult refugees were randomly selected from a total of 14,000 refugees living in the camp and assessed for eligibility. The selection was conducted by using a computer-generated random number list. 688 refugees met the criteria and 45 were randomly selected and approached who were not actively seeking treatment. | PTSD symptoms (IES-R), depression (BDI-II) | EMDR group had significantly lower trauma scores at posttreatment as compared with the wait-list group (d= 1.78, 95% CI: 0.922.64). The EMDR group also had a lower depression score after treatment as compared with the wait-list group (d=1.14, 95% CI: 0.351.92) | 1 | 7 app, 0 non-app, 1 no info | 40 | 38 |
| [Ponguta et al](https://onlinelibrary.wiley.com/doi/pdf/10.1002/cad.20314), 2019 ^67^ | Lebanon | Mother-child dyads of Palestinian and Syrian refugees, and low SES Lebanese families | MOCEP  (25, II) | WLC | N/A | 106 | *Convenience Sampling*  Mothers were recruited from the catchment areas of the partner NGOs. After completing a baseline questionnaire, mothers were randomized to either the intervention or wait-list control group. | Implementation evaluation:  Exploration of context,  Participation,  Attendance,  Program quality,  Engagement of maternal participation,  Self-reported impacts,  Enablers of and barriers to the program’s evaluation | Authors provided evidence to highlight contextual characteristics that should be considered in future program adaptations and applications, including inter-ethnic relations, concerns around security and trust and the importance of engaging community leaders in the dissemination of information about a program’s intent. Despite multiple challenges, implementation and robust evaluations of early childhood parenting programs in fragile contexts are feasible and urgently needed. | 0 | 7 app, 0 non-app, 1 no info | 39 | 39 |
| [Bryant et al](https://journals.plos.org/plosmedicine/article?id=10.1371/journal.pmed.1003949), 2022 ^68^ | Jordan | Camp-based Syrian refugees | gPM+  (5, II) | EUC | 624 | 410 | *Purposive sampling*  Recruitment was conducted by Arabic-speaking assessors through door-to-door screening of consecutive caravans; to reduce contamination of the interventions, only 1 adult per caravan was invited to participate in the study. It was considered that there would likely not be significant contamination between neighbouring caravans because socialization did not adhere to caravan proximity. The invitation was initially extended to the person who answered the door, and if they declined, the offer was made to another adult living in the caravan. Informed consent involved 2 steps: (1) consent to participate in the screening; and (2) participants who screened positive were invited to provide their consent to participate in the trial. | Anxiety and depression, functional impairment, PTSD symptoms, personally identified problems, prolonged grief symptoms, prodromal psychotic symptoms, parenting behaviour, children’s self-reported mental health  (HSCL-25, WHODAS 2.0, PCL-5, PSYCHLOPS, PG-13, PQ-B, APQ, PSC) | At follow-up, participants in gPM+ showed greater reduction on HSCL depression scale than those receiving EUC (mean difference, 3.69 [95% CI 1.90 to 5.48], p=.001; effect size,0.40). There was no difference between conditions in anxiety (mean difference −0.56, 95% CI− 2.09 to 0.96; p=.47; effect size, −0.03). Relative to EUC, participants in gPM+ had greater reductions in severity of personally identified problems (mean difference 0.88, 95% CI 0.07 to 1.69; p=.03), and inconsistent disciplinary parenting (mean difference 1.54, 95% CI 1.03 to 2.05; p<.001). There were no significant differences between conditions for changes in PTSD, disability, grief, prodromal symptoms, or childhood mental health outcomes. | 3 | 7 app, 0 non app, 1 no info | 41 | 36 |
| [Acarturk et al, 2022](https://link.springer.com/article/10.1186/s12888-021-03645-w) ^69^ | Turkey | Syrian refugees | Pilot gPM+  (5, III) | ECAU | 78 | 46 | *Purposive sampling*  The project was conducted in collaboration with RASASA in Turkey, an NGO which provides health, psycho-social and legal support to Syrians in need. The study was implemented in Sultanbeyli, a suburb of Istanbul which hosts more than 30,000 Syrian refugees. Participants were recruited via dissemination of brochures and posters in RASASA to its beneficiaries, advertisement through social networking platforms and referrals from RASASA’s health and social support workers. Syrian refugees living in Sultanbeyli who gave their consent to participate were screened to assess whether they were eligible to participate in the study. | Anxiety and depression, PTSD symptoms, personally identified problems, economic impacts (health service utilisation and lost, employment productivity, lifetime trauma exposure  (HSCL-25, PCL-5, PSYCHLOPS, CSRI, PMLDC) | There were no barriers experienced in recruiting study participants and in randomising them into the respective study arms. Retention in gPM+ was high (75%). Qualitative analyses of the interviews with the participants showed that Syrian refugees had a positive view on the content, implementation and format of gPM+. No adverse events were reported during the implementation. The study was not powered to detect an effect. No significant difference between gPM+ and E‑CAU group on primary and secondary outcome measures, or in economic impacts were found. | 3 | 7 app, 0 non app, 1 no info | 43 | 34 |
| [Sieverding et al](https://www.ghspjournal.org/content/ghsp/10/1/e2100079.full.pdf), 2022 ^70^ | Lebanon | Syrian refugee girls aged 11–14 years and mothers | AMENAH  (8, II) | No comparison group | 340 | 203 | *Purposive sampling*  1-Obtain lists of the names and birthdates of Syrian female students from the 4 partner schools – Role: Research team in coordination with schools (Location: Schools), 2-Send letter about the study to parents of all girls aged 11–14 years, informing them about the study and inviting them to attend a meeting on school premises – Role: Research team in coordination with schools (Location: Schools), 3-Hold a meeting in each school to describe the study and obtain preliminary consent from attending parents to be visited at home; record parents’ contact information – Role: Research team and community workers, in coordination with schools (Location: Schools), 4-Conduct home visits to obtain the consent of parents to enrol their daughter in the pilot intervention – Role: Community workers (Location: Home visits), 5-Obtain assent from girls to participate in Amenah – Role: Research team (Location: Community partner (MAPs) premises) | Increased school retention and later ages at marriage, program reach, fidelity, dose received | Process evaluation results showed that socio-demographic factors predicted attendance among mothers, but relationships with peers in the intervention were the only significant predictor of attendance among girls. The primary outcomes of the pilot were attitudinal measures related to education and marriage. Attitudes toward education were highly positive at baseline and did not change over the course of the intervention. There were no significant changes in girls’ ideal age at marriage. Among girls aged 13 and older at endline, the mean self-reported expected age at marriage increased slightly from 20.2 to 20.8 years (P<.05). Results also suggest that girls may ad-just their expected age at marriage downward as they become older and if they drop out of school. | 0 | 5 app, 1 non-app, 2 no info | 41 | 35 |
| [Bunn et al](https://www.tandfonline.com/doi/pdf/10.1080/01933922.2021.2000084), 2022 ^71^ | Jordan | Syrian refugees | Multimodal: Group counselling + Physiotherapy  (10 each, III) | No comparison group | N/A | 31 | *Purposive Sampling*  Recruitment took place at the clinic locations in Amman and Zarqa serving urban refugees. Recruitment was limited to Syrian men and women, aged 21 years and older, who had already participated in the interdisciplinary group intervention and had completed a 3-month follow-up assessment. A purposeful sampling approach was employed to identify a demographically varied sample according to gender, age, and clinic location (Patton, 2015). Recruitment activities were limited to participants who expressed interest in participating in research. The partner organization conducted an initial screening call to confirm interest in participating in research and willingness to be contacted by the researcher. Following this, contact information of prospective study participants was shared with the researcher. Prospective participants were contacted by the research assistant and invited to participate in an interview. | 1) Gaining perspective, increased hope  2) Recreating social spaces  3) Better coping skills  4) Gaining confidence  5) Grieving loss of roles/identities: Some men grieved the loss of their role as breadwinner  6) Family dynamics improved. | Drawing from grounded theory analytical methods, a core relational process, *sharing stories eases pain*, was derived from the data to explain how group members came to develop close, caring relationships, which led to other psychosocial benefits. The findings fill a gap in the group treatment literature specific to refugees and advance understanding of group relationships as an active ingredient promoting change. Findings also suggest that group-based treatment may be uniquely suited to address some of the broader social-relational consequences of conflict and forced migration | 3 | 3 app, 3 non-app, 2 no info | 39 | 37 |
| [Sahyoun et al](https://bmcpublichealth.biomedcentral.com/articles/10.1186/s12889-019-7950-3), 2019 ^72^ | Lebanon | Camp-based Palestinian refugee women | Healthy Kitchens, Healthy Children  (60+, II) | No comparison group | 51 | 32 | *Purposive Sampling*  With the help of UNRWA’s Relief and Social Services office, Palestinian women living in the camps were identified and contacted by social workers and CBO staff to participate in this intervention. Social workers and CBOs reached out to women who had either applied for the UNRWA social safety net program, or had attended previous CBO activities (language literacy, computer literacy and hairdressing classes), and had previously expressed a need/willingness to work. | Economic wellbeing, food security, decision making, reported health (physical and mental), social support | Participating women had a 13% increase in household expenditure. This was translated into a significant increase in food (p< 0.05) and clothing expenditures (p< 0.01), as well as a reduction in food insecurity score (p< 0.01). These findings were supported by qualitative data which found that the kitchens provided women with financial support in addition to a space to form social bonds, discuss personal issues and share experiences. | 0 | 5 app, 1 non-app, 2 no info | 37 | 38 |
| [Miller et al](https://www.sciencedirect.com/science/article/pii/S0145213420301678), 2020 ^73^ | Lebanon | Caregivers: Syrian refugees, Palestinian refugees, Lebanese | CSI pilot  (9, II) | WLC | 151 | 151 | *Purposive Sampling*  Participants were recruited into the study in collaboration with the local community organizations that hosted the intervention and assessments. Specific methods included community breakfasts to announce the study, door to door recruitment in target communities, visits by outreach staff to settings where men commonly gather, and word of mouth | Stress, Psychosocial Wellbeing, Psychological distress, stress management, Parenting, Warmth-responsiveness, harsh parenting, parenting knowledge, IC psychosocial wellbeing parent and child reports | Randomization was successful, retention was high (99 %), as was intervention completion (95 % among women, 86 % among men). Implementation fidelity was excellent. Blinding was largely, though not completely effective. The CSI group showed significantly increased parental warmth and responsiveness, decreased harsh parenting, lowered stress and distress, improved psychosocial wellbeing, and improved stress management. CSI parents reported increased child psychosocial wellbeing. Control families showed no significant change on any variable. | 0 | 7 app, 1 non-app, 0 no info | 36 | 38 |
| [Yurtsever et al](https://www.ncbi.nlm.nih.gov/pmc/?term=10.3389/fpsyg.2018.00493%5BDOI%5D), 2018 ^74^ | Turkey | Camp-based Syrian refugees | EMDR G-TEP  (2, IV) | Control group | 97 | 47 | *Purposive Sampling*  Five therapists at the camp gave seminars about “war and trauma” at schools and leisure centres of the camp. The study and the therapy program were announced at several locations at the camp by the school and leisure centre personnel in early September. Clinical staff at the Psychosocial Support Centre within the camp identified potential participants who met the study inclusion criteria. | PTSD symptoms (IES-R), depression (BDI-II) and mental disorders (MINI) | Results showed that the EMDR G-TEP group had significantly lower PTSD and depression symptoms after intervention. The percentage of PTSD diagnosis decreased from 100 to 38.9% in the EMDR G-TEP group and was unchanged in the control group. Following the EMDRG-TEP intervention 61.1% of the experimental group no longer had a PTSD diagnosis; this decrease was maintained at 4 weeks follow-up. | 1 | 6 app, 0 non app, 2 no info | 39 | 34 |
| [AKV Hansen et al](https://tidsskrift.dk/torture-journal/article/view/103977/152916), 2017 ^75^ | Jordan, Palestine, Egypt, Lebanon, Iraq, Tunisia, Libya, Sudan, Syria | Arabic mental health professionals (Training of the trainer) + torture survivors (NET participants) | NET  (Variable, Multi-layer) | No comparison group | N/A | 110 | *Purposive Sampling*  Torture survivors who were clients of the rehab centres in the MENA region were recruited by the trained MH professionals. | PTSD (HTQ), Anxiety-depression (HSCL-25), disability and functioning (WHODAS 2.0), Single item question assessing pain levels, written personal narratives or experiences. | Findings show a statistically significant reduction in average psychological symptom load for PTSD (from 3.20 to 1.80), anxiety (2.78 - 1.61) and depression (2.75 - 1.96) with the largest effect on PTSD symptoms, and a larger effect for women than men. The results indicate improvements in self-reported health (3.85—2.82) and physical disabilities (2.90—1.76), as well as reduction in pain perceptions after therapy (4.44-3.44). The duration of treatment was three months on average with a span from one to eight months. | 4 | 3 app, 1 non-app, 4 no info | 42 | 31 |
| [Acarturk et al, 2022](https://onlinelibrary.wiley.com/doi/pdfdirect/10.1002/wps.20939) ^76^ | Turkey | Syrian refugees | Self-Help Plus  (5, III) | ECAU | 1186 | 642 | *Purposive Sampling*  Local non-governmental organizations (NGOs) implementing projects for refugees in Turkey were approached to identify potentially eligible participants. These NGOs provide integrated reception services that include food, housing; legal, educational, health care and social guidance and support; and programs to promote socioeconomic inclusion and integration. Participants were consecutively invited to participate by members of the re-search team, in agreement with local service staff, who facilitated contacts. | Current mental disorders (MINI), psychological distress (GHQ-12), PTSD symptoms (PCL-5), depression (PHQ-9), personally identified psychological outcomes (PSYCHLOPS), functional impairment and subjective wellbeing (WHO-DAS, WHO-5), general health (EQ-5D-3L), adverse life events (HTQ, PMLD) | Self-­Help Plus participants were sig­nificantly less likely to have any mental disorders at six-month follow­ up compared to the ECAU group (21.69% vs. 40.73%; Cramer’s V=0.205, p<0.001, risk ratio: 0.533, 95% CI: 0.408­0.696). Analysis of secondary outcomes suggested that Self Help Plus was not effective immediately post­intervention, but was associated with beneficial effects at six-month follow-up in terms of symptoms of depression, personally identified psychological outcomes, and quality of life | 6 | 8 app, 0 non app, 0 no info | 38 | 34 |
| [James et al](https://www.mdpi.com/1660-4601/18/21/11674), 2021 ^77^ | Lebanon/Malaysia (Rohingya) | Syrian refugees (Lebanon) and Rohingya refugees (Malaysia) | a) IPV workshop  b) IPV poster campaign  (3 / 1, II) | Control group | N/A | 148  500 | *a) Purposive Sampling:*  Syrian community member participants in Lebanon were primarily sampled from the El Marj community in Bekaa Valley. Potential participants were recruited by phone or in person at partner organization activities by Syrian and Lebanese members of the team, using a recruitment script. In both settings, for safety reasons, only one member of a couple or household was invited to participate. Those who expressed interest participated in a verbal informed consent process in a private area.  b) *Cluster comparison*: Communities were randomized to condition, reducing the likelihood of contamination resulting from neighbours talking to each other about the campaign. | a) IPV prevalence and acceptability, beliefs about gender relations, attitudes toward help-seeking, mental health (RHS-15), Functional impairment, social cohesion, self-efficacy, community-efficacy, coping (COPE), perceived workshop impact, workshop satisfaction, reactions to research participation (RRPQ)  b) acceptability of violence, individual beliefs about gender relations, relationship problem-solving efficacy, help-seeking personal and beliefs | Women in both settings found IPV less acceptable in the poster condition. Help-seeking preferences were also influenced by the poster for women and men in both countries. | 0 | 5 app, 0 non app, 3 no info | 35 | 37 |
| [Sim et al](https://journals.sagepub.com/doi/full/10.1177/1049731520953627), 2021 ^78^ | Lebanon | Syrian refugees | Families Make the Difference- parenting  (10, II) | No comparison group | 714 | 254 | *Purposive Sampling*  Participants were recruited by local IRC staff through community and school outreach visits and referrals from IRC case management staff, other nongovernmental agencies, and com-munity members | Child maltreatment (MICS), parental behaviour (PARQ), child psychosocial problems (SDQ, SCARED, SMFQ), parental mental health (DASS-21, PTSD checklist), mother’s perceived social support (mMOS-SS) | Results showed significant reductions in harsh punishment and rejecting parenting behaviour and significant improvements in measures of parental and child mental health from pre- to postintervention. On average, parents completed 7.7 of 10 sessions | 0 | 5 app, 1 non app, 2 no info | 34 | 37 |
| [Akhtar et al](https://www.tandfonline.com/doi/pdf/10.1080/20008198.2021.1932295), 2021 ^79^ | Jordan | Camp-based Syrian refugees | gPM+  (5, II) | ETAU | 207 | 55 | *Purposive Sampling*  Arabic-speaking assessors conducted recruitment through door-to-door screening of consecutive caravans; to reduce contamination of the interventions, only 1 adult per caravan was invited to participate in the study. | Psychological distress (HSCL-25), prolonged grief (PH-13), prodromal psychotic symptoms (PQ-B), paediatric symptoms checklist, traumatic events exposure (TEC), post-migration living difficulties (PMLD) | Of the 207 persons screened, 64 (31%) screened positive for psychological distress. Of the 35 randomized into the Group PM+ intervention, 24 (69%) completed the intervention. No adverse events were reported throughout the trial. Children whose parents received Group PM+ had greater reductions in internalizing and externalizing symptoms at posttreatment. 55 (86%) participants completed the post-assessment follow-up. These results demonstrate both the feasibility of conducting the trial in a camp and acceptance of the Group PM+ intervention by Syrian refugees | 0 | 7 app, 1 non app, 0 no info | 35 | 36 |
| [Powell et al](https://journals.sagepub.com/doi/pdf/10.1177/00207640221074808), 2023 ^80^ | Jordan | Jordanians and resettled Syrian refugees | Mental health awareness  (4, II) | No comparison group | N/A | 21 | All individuals who took part in the study were recruited into the study through their doctors’ recommendations. Fliers were posted at the health clinic to inform individuals of the study.  *Maximum Variation Sampling* was used to obtain perspectives from a heterogenous pool of participants. The sample was drawn from a larger pool of participants from the longitudinal quantitative study of the HCC-MH intervention. Based on pre-identified dimensions of variation, the researchers recruited a sample of Syrian and Jordanian men and women who participated in the HCC-MH intervention. At the end of the final session, participants were informed of the qualitative evaluation by the facilitator and invited to participate in the focus group discussions. | Focus group discussions to explore benefits of mental health awareness intervention. Four intervention sessions:  Session 1: A Healthier Emotional You  Session 2: How Stress Affects the Body  Session 3: Understanding and Coping with Grief  Session 4: Coping Techniques for a Healthier You | Six central themes emerged from the data including: (1) awareness; (2) behaviour changes; (3) reduction in stigma; (4) connecting physical and mental health; (5) relationships; and (6) coping. Notably, female participants stated participation in the intervention fostered awareness of their own emotional needs, which led to positive lifestyle and behaviour changes. Participants also described how the information provided in the intervention normalized emotional distress and aided understanding of the interconnection between physical and mental health. The amplification of healthy coping strategies to reduce stress and distress was also a prominent theme. | 0 | 4 app, 3 non-app, 1 no info | 33 | 36 |
| [Cuijpers et al](https://journals.plos.org/plosmedicine/article?id=10.1371/journal.pmed.1004025), 2022 ^81^ | Lebanon | Syrian refugees | Step-by-Step, Online  (5, III) | ECAU | 1380 | 569 | *Purposive Sampling*  Recruitment of participants took place through advertising for the research project on several social media platforms, by posting and boosting posts on the official social media pages of the National Mental Health Programme on Facebook and Instagram. Additionally, outreach methods took place with the network of NGOs and UN agencies taking part in a mental health and psychosocial support taskforce whereby meetings were held in different regions with the Syrian community to introduce the project, followed by WhatsApp broadcasts that were sent by the organizations to their Syrian beneficiaries.  Interested participants could access the web version or download the mobile app, where information was given about the intervention and the study, including an animated video explaining key points. After completing informed consent and the baseline self-screening instruments, participants who met inclusion criteria were asked to complete additional baseline questionnaires. | Depression (PHQ-9), impaired functioning (WHODAS), subjective well-being (WHO-5), anxiety (GAD-7), post-traumatic stress (DSM-5), and self-described problems (PSYCHLOPS). | Intention-to-treat (ITT) analyses showed intervention effects on depression (standardized mean differences [SMDs]: 0.48; 95% CI: 0.26; 0.70; p < 0.001), impaired functioning (SMD: 0.35; 95% CI: 0.14; 0.56; p < 0.001), post-traumatic stress (SMD: 0.36; 95% CI: 0.16; 0.56; p < 0.001), anxiety (SMD: 0.46; 95% CI: 0.24; 0.68; p < 0.001), subjective well-being (SMD: 0.47; 95% CI: 0.26; 0.68; p < 0.001), and self-identi- fied personal problems (SMD: 0.49; 95% CI 0.28; 0.70; p < 0.001). Significant effects on all outcomes were maintained at 3 months follow-up. During the trial, one serious adverse event occurred, unrelated to the intervention. The main limitation of the current trial is the high dropout rate. | 3 | 6 app, 1 non-app, 1 no info | 36 | 32 |
| [Damra et al](https://vc.bridgew.edu/jiws/vol23/iss1/33/#:~:text=The%20results%20indicated%20that%20the,the%20environmental%20sub%2Ddimension).), 2022 ^82^ | Jordan | Syrian refugee abused women | Psychodrama  (12, II) | UC | 116 | 40 | *Purposive sampling*  All refugee women who visited the Woman Health Center (WHC) in Al –Zarqa Governorate between the 21st of September 2016 and the 30th of March 2017 and matched the study eligibility criteria were assessed to participate in this study.  All recognized women as IPV victims were invited to participate in the study. Through the plenary session, the research assistants explained the study's primary purpose, procedures, and ethical considerations for all women (89 women out of 116 attended this plenary session). | IPV (DVQ), QoL (WHOQoL-100) | Results indicated that the psychodrama group participants demonstrated a statistically significant decrease in IPV severity and enhancement in all QoL sub-dimensions (with one exception of the environmental sub-dimension). | 0 | 4 app, 0 non-app, 4 no info | 32 | 32 |
| [Veale et al, 2019](https://cora.ucc.ie/server/api/core/bitstreams/e9863e34-2a29-44ad-8841-b11b99214d10/content) ^83^ | Lebanon | Syrian refugee + Lebanese men | Engaging Men  (12, II) | No comparison group | N/A | 1469 | *Purposive Sampling*  Communities were informed about the course through radio announce- ments that broadcast a contact phone number for the lead facilitator. Facilitators met with focal community people such as the Mukhtar and municipality staff, spoke about the project as being about gender, violence and nonviolence with funding for a community project. These focal people recruited groups of men known to them; one facilitator explained how some of these focal people ‘were leaders, old men, they talk about how in Syria they support each other and give to people from community, as part of a Muslim community.’ In these ways, groups formed organically in response to different public calls. | Promoting peaceful interactions with others, reducing violence and gender-based violence, child protection and caregiving, and increasing community safety and harmony through a community project. | Focus group discussions were conducted with 130 men, 28 wives, and 17 children of male participants, 10 family visits and 20 individual interviews by peer researchers. The findings were that programmes facilitated a safe emotional space for men to meet collectively to talk about their problems, to become more attuned and reflective about their relationships with their wives and children, which resulted in improved patterns of interaction and communication, increased openness and greater tolerance by men of changing gender roles in families as a result of displacement. | 0 | 3 app, 1 non app, 4 no info | 31 | 33 |
| [Lakkis et al](https://www.frontiersin.org/articles/10.3389/fpsyt.2020.00257/full), 2020 ^84^ | Lebanon + Jordan | Camp-based Syrian refugee parents | ECD pilot  II | No comparison group | 125 | 67 | *Purposive Sampling*  The directors of community and/or social service centres (identified as community leaders) located within each camp facilitated the recruitment process under the supervision of ARC and PI research coordinators (n = 4). The research coordinators invited the eligible parents to participate in this study. | Well-being (WHO-5), Parenting stress (PS-SF), psychosocial problems and strengths in child’s daily life (SDQ), Parental discipline strategies (DSQ) | Parents’ mental health and wellbeing improved (p < 0.001, Cohen’s d: 0.61) and their parenting index score was reduced (p < 0.001, Cohen’s d: 1.24). Some of their dysfunctional interactions with their children as well as the perceived difficulties and conduct problems in their children aged 3 to 6 years were also reduced significantly. | 0 | 7 app, 1 non-app, 0 no info | 31 | 33 |
| [Lilleston et al](https://academic.oup.com/heapol/article/33/7/767/5037237), 2018 ^85^ | Lebanon | Syrian refugee women | GBV mobile service delivery  (variable, Multi-layer) | Control group | N/A | 50 | The community mobilizers engaged the community in the program through a cadre of outreach activities including meetings with community leaders, ‘tea and coffee’ information sessions with community members and door-to-door visits. The mobile teams additionally identified female ‘focal points’ among the Syrian refugee population in each community whose role was to engage community members, share information about the mobile services and provide referrals for GBV survivors. | The program served 25 different communities, conducting 283 site visits in which over 1000 PSS activities and 100 community mobilization activities were implemented. During this time, caseworkers saw 50 unique clients regarding 56 different GBV incidents.  Key outcomes: safe spaces, community engagement, safe referrals, survivor-centred approach, confidentiality, accessibility of services. | In-depth interviews with IRC staff (n= 11), Syrian refugee women (n= 40) and adolescent girls (n= 26). Findings suggest that by providing free, flexible service delivery in women’s own communities, the mobile model overcame barriers that limited women’s and girls’ access to essential services, including transportation, checkpoints, cost and gendered expectations around mobility and domestic responsibilities. Participants described the services as strengthening social networks, reducing feelings of idleness and isolation, and increasing knowledge and self-confidence. Results indicate that the model requires skilled, creative staff who can assess community readiness for activities, quickly build trust and ensure confidentiality in contexts of displacement and disruption. Referring survivors to legal and medical services was challenging in a context with limited access to quality services. | 0 | 3 app, 3 non-app, 2 no info | 28 | 36 |
| [Blackwell et al](https://web.s.ebscohost.com/ehost/pdfviewer/pdfviewer?vid=1&sid=0b39cbea-beb5-42df-8a70-21582e1a718b%40redis), 2022 ^86^ | Kurdistan, Iraq | Displaced Iraqi, Syrian and Kurdish people | CBT by local therapists  (12, IV) | No comparison group | N/A | 28 | Convenience sample of 18 years or older Arabic speakers seeking therapeutic services at Jiyan Foundation at three locations in the autonomous Kurdistan Region. Arabic speakers seeking services signed a consent to participate in a study during treatment but were told not participating or withdrawing later would not impact services. | Exposure to traumatic events (HTQ), PTSD symptoms (DSM-5), depression (PHQ-9) | Both PTSD scores (b = 9.87, SE = 1.37, d = 1.74, p , .001) and depression scores (b = 2.75, SE = .47, d = 1.32, p , .001) decreased with a large effect. | 0 | 4 app, 1 non-app, 3 no info | 29 | 34 |
| [Jirmanus et al](https://www.sciencedirect.com/science/article/pii/S0277953621000319), 2021 ^87^ | Lebanon | Dom, Lebanese, and Syrian refugees | CBPR trash collection  (0, II) | No comparison group | N/A | 83 | The researchers collaborated with a local NGO to recruit women who frequently participated in community education activities to the community advisory board (CAB) who were from the three social groups (Lebanese, Dom and Syrian refugees). NGO staff informed married women with children in the neighbourhood about the study and CAB members encouraged their neighbours to participate in focus groups. | Researchers applied Community Based Participatory Research (CBPR) methodology in collaboration with an NGO in an underserved Beirut neighbourhood to address health inequities, build social cohesion among refugees and host populations, and empower community members to develop a community health intervention. | The trash collection initiative failed to take place and impact change due to several structural challenges from entrenched local and national hierarchies, ineffective political processes, and inter- and intra-group conflict driven by competition over privatized social services. To expedite change in the community, researchers found empowering powerful local individuals rather than oppressed community participants was more effective. | 0 | 3 app, 2 non app, 3 no info | 28 | 35 |
| [Budosan et al](https://academicjournals.org/journal/INGOJ/article-full-text/727C48058926), 2016 ^88^ | Turkey | Urban Syrian refugees | Multi-level MHPSS delivery: i) MHPSS training, ii) MH intervention,  iii) Social intervention  (Variable, multi-layer) | No comparison group | N/A | i)N/A  ii) 1163, 151  iii) 1196 | Direct beneficiaries were selected by the NGOs running the interventions based on eligibility criteria through the health centres. | Well-being, resilience | The results showed improvement of resilience and well-being of the targeted population. The intervention was effective in an urban context. The intervention filled existing gaps in the provision of MHPSS services to urban Syrian refugee population in Kilis and it was well-integrated within the local health/social welfare system and Syrian refugee community. Although, it did not target some needs/daily stressors of refugees which ranked high according to the HESPER survey (income & livelihood, place to live in), it increased resilience of beneficiaries and helped them to better cope with existing stressors. | 0 | 4 app, 1 non-app, 3 no info | 37 | 26 |
| [Talhouk et al](https://www.tandfonline.com/doi/pdf/10.1080/13549839.2021.1973393), 2021 ^89^ | Lebanon | Syrian refugee women | Vertical gardening  (20, II) | No comparison group | 51 | 44 | *Purposive sampling*  Consultations were made with international aid organisations and the Lebanese ministry of social affairs to identify potential intervention sites. According to on-the-ground government and international aid agencies, four displaced Syrian communities in North Lebanon expressed willingness to learn more about the project. Researchers visited women in every family in the study sites, explained the purpose of the project and invited them to join the introductory project session. | Depression (BDI-II), perceptions of gardening | Participants were less depressed at the end of the gardening programme, with depression scores significantly lower than preparatory phase scores. Group interviews were also conducted to assess women’s perception of gardening halfway through the intervention phase. Women’s engagement in gardening activities was also reflected by estimating yields. Participants indicated that they joined the programme because they saw it as a stress relief activity, they were interested to learn about vertical gardening, they enjoyed the aesthetic value of plants, they wanted to produce food and they felt that gardening provided an opportunity to socialise. | 1 | 6 app, 2 non-app, 0 no info | 28 | 34 |
| [Lancaster et al](https://www.tandfonline.com/doi/pdf/10.1080/10615806.2020.1773446), 2020 ^90^ | Iraq | Camp-based Kurdish Iraqi internally displaced persons | GROW Resilience  (8, II) | No comparison group | 1129 | 766 | *Purposive Sampling*  Recruitment for the GROW intervention within each IDP camp was done systematically. That is, as each IDP camp is divided into sectors, the recruitment team, who were paid staff of Tutapona, was able to go through each camp sector by sector recruiting participants. Starting in one sector, they proceeded tent to tent describing the program and inviting people to participate until they were through that sector. Then they would go to the next sector. People from the same sector within the camp would participate together to help build relationships and community within that sector. Once the program was more established within the camps, organization staff created a waiting list, and referrals were made by other organizations for individuals in significant need. | PTSD symptoms (SPTSS) | Results indicate significant decreases in symptoms of PTSD for participants from pre- to post-intervention and demonstrates these changes were maintained at a three-month follow-up. Post hoc results indicated participants who were older, female, or who attended more sessions were more likely to respond to the intervention. | 3 | 6 app, 1 non-app, 1 no info | 29 | 31 |
| [Yassin et al](https://link.springer.com/article/10.1007/s10903-017-0657-6), 2018 ^91^ | Lebanon | Camp-based Palestinian refugees | Integrated MH program  (Variable, multi-layer) | No comparison group | N/A | 28 | Not reported | The mental health program was integrated into primary healthcare and ran for 3 years, where 28 patients participated in the focus groups to evaluate the program, alongside providers and stakeholders. | The program provided easy access, good quality care, decreased stigma, as perceived by participants, and revealed a sense of community contentedness. In addition, several short-term outcomes were achieved, such as increasing the numbers of patients visiting the centre/ receiving mental health treatment. However, lack of planning for sustainability and proper procedures for hand-over of the program constituted a major downfall. Program discontinuation posed ethical dilemmas, common in provisional interventions in underprivileged refugee communities. | 0 | 2 app, 3 not app, 3 no info | 28 | 31 |
| [Bruno et al](https://www.researchgate.net/profile/Akiko-Kitamura/publication/333221618_Assessment_of_mental_health_and_psycho-social_support_pilot_program%27s_effect_on_intended_stigmatizing_behavior_at_the_Saftawi_Health_Center_Gaza_a_cross-sectional_study/links/5ce2d49d92851c4eabb155fb/Assessment-of-mental-health-and-psycho-social-support-pilot-programs-effect-on-intended-stigmatizing-behavior-at-the-Saftawi-Health-Center-Gaza-a-cross-sectional-study.pdf), 2019 ^92^ | Gaza/  Palestine | Gaza refugees | Integrated MHPSS program  (Variable, multi-layer) | Control group – Saftawi (int) and Nasser (control) health centres | N/A | 408 | Both health centres are in the Gaza Strip and operated by UNRWA with demographically similar populations. Between June and July of 2017, 205 attendants at Saftawi Health Centre (intervention) and 203 at Nasser Health Centre (control) were randomly selected and asked to take short research survey. The random sample was generated systematically by selecting every fifth client entering the health centre (HC). To ensure equal participation between genders, if 2 members of the same gender were randomly picked in succession, the second would be skipped to the next fifth client to choose the other gender. | Stigmatising behaviour (RIBS) | Saftawi respondents endorsed significantly less intended stigmatizing behaviour compared to Nasser respondents (p<0.001). Multivariable analysis demonstrated significantly less intended stigmatizing behaviour at Saftawi compared to Nasser (p<0.01) while controlling for demographic covariables. | 12 | 4 app, 1 not app, 3 no info | 23 | 35 |
| [Ruggeri et al](https://www.nature.com/articles/s41599-021-00784-z), 2021 ^93^ | Lebanon | Lebanese nationals, Syrian  refugees and Palestinians | Nudges and boosts for decision-making, online  (1, II) | Control group | 754 | 741 | All data were collected via the online survey platform Qualtrics using a paid collection service with offices and field staff in Lebanon. All aspects of the protocol were administered online in Arabic. Lebanese participants accessed the instrument through an online portal, whereas refugees (N = 244) had to be approached in person due to geographic restrictions or no internet access | Subjective well-being, risk-taking, trust | Higher subjective well-being was associated with more risk-taking among refugees (β = 0.07, SE =0.02, z = 4.63, p < 0.01), but not among the Lebanese host population (β =−0.003, SE = 0.01, z = −0.32, p = 0.75). However, average subjective well-being did not significantly differ between the Lebanese host population and refugees (absolute difference= −1.27, 95% CI = [−2.83, 0.29], on a 60-point scale), or between Syrian and Palestinian refugees (absolute difference = −1.53, 95% CI = [−4.16, 1.08]). Behavioural interventions (nudges and boosts) designed to support people in making choices more advantageous for them showed moderate effects. There is a clear pattern of greater risk-taking for refugees with better subjective well-being. This is an important finding as greater risk-taking can be associated with several negative health outcomes, particularly in vulnerable populations. | 0 | 4 app, 1 non app, 3 no info | 27 | 30 |
| [Khoury & Daouk](https://academic.oup.com/jrs/article/35/1/662/6375742), 2021 ^94^ | Lebanon | Syrian refugee women and Lebanese women | Group PSS  (12, II) | No comparison group | N/A | Pre 85  Post 71 | Health workers were recruited from organizations who are partners with UNFPA and who recommended them. The health workers were mostly social workers, psychologists, counsellors, nurses, and community health workers and trained to run the PSS program. Trained health workers announced the launching of the groups in the centres where they worked and informed their staff, and thus, the groups were made from participants who were interested in the program. | Anxiety-depression (HSCL-25) | Results showed that levels of anxiety and depression seemed to decrease significantly after the intervention. An additional outcome was that most of the participants felt supported by other women. | 0 | 5 app, 2 non-app, 1 no info | 33 | 22 |
| [Womersley et al](https://www.researchgate.net/profile/Gail-Womersley-2/publication/330608527_Collective_trauma_among_displaced_populations_in_Northern_Iraq_A_case_study_evaluating_the_therapeutic_interventions_of_the_Free_Yezidi_Foundation/links/5d4158c4a6fdcc370a6f21f8/Collective-trauma-among-displaced-populations-in-Northern-Iraq-A-case-study-evaluating-the-therapeutic-interventions-of-the-Free-Yezidi-Foundation.pdf), 2019 ^95^ | Iraq | Yezidi women | MHPSS services delivery  (Variable, multi-layer) | No comparison group | 200 | Pre 170  Post 113 | The Free Yezidi Foundation women’s centre, inside the Khange IDP camp, serves all the women in the camp, including women who have escaped ISIS captivity. The project collaborates with over twenty non-governmental and governmental actors in Kurdistan to coordinate MHPSS responses. | Well-being (WHO-5), PTSD symptoms (HTQ), baseline prevalence rates of trauma, culturally informed subjective experiences of trauma within this specific socio-political context, and lessons learnt from the implementation of this psycho-therapeutic intervention. | Results of the WHO-5 indicated a 74% increase in self-reported well-being among service users who completed the programme. Baseline prevalence rate of posttraumatic stress disorder was 81.25%, which decreased to 45% upon completion of the programme. Qualitative analysis of interviews and FGDs highlighted that a significant impact on mental health were collective, multiple losses and separations (including family members who sought refuge abroad), the fact that not all Yezidi held in captivity have returned, fear of ongoing attacks and daily stressors related to poor living conditions. The client satisfaction questionnaire revealed a 91% level of satisfaction with the project. | 0 | 5 app, 1 non app, 2 no info | 27 | 27 |
| [Sakhi et al](https://www.nap1325.nl/assets/PDF/Intervention20158-4389962_121139-3-v2.pdf), 2022 ^96^ | Lebanon | Camp-based refugee women (Syrian, Palestinian, Lebanese) | Drama therapy  (12, II) | No comparison group | Intensive  Program: Not reported  Follow-up Program:  Not reported | 64  41 | *Purposive Sampling*  The program coordinator in Lebanon contacted partner NGOs who have centres in Shatila camp and coordinated the implementation process including NGO centres that have previously delivered cultural and MHPSS programmes, have direct contact with potential beneficiaries and were able to help in the recruitment of potential beneficiaries. The social workers of the centres recruited participants that were adult women above 18 years in need of psychological support and created a list of names and phone numbers of the interested women to be passed on to the program coordinator. | Improve emotional awareness and self-awareness, create a sense of belonging, tackle negative psychological symptoms | The impact of the programmes is shown through compiled qualitative findings from 59 interviews, 4 FGDs and subsequent programme reports. Findings include emotional regulation, benefits of movement and exercise, a sense of belonging and changes within the family after the intervention. Drama therapy can be an effective psychological intervention for refugee women as it provides inclusive care and avoids stigma. | N/A | 3 app, 3 non app, 2 no info | 30 | 23 |
| [Al-Rousan et al](https://www.tandfonline.com/doi/pdf/10.1080/13623699.2018.1518748), 2018 ^97^ | Jordan | Camp-based Syrian refugee university students | Higher education tuition + stipend  (N/A, III) | Control group | N/A | 98 | Not reported | Subjective experience of peace across 7 domains (PEACE), benefits and limitations of the scholarship programme the impact of the programme on their families and themselves, recipients’ outlook on the future, persistent obstacles faced in spite of sponsorship and suggestions for additional helpful supports. | The overall mean PEACE score among the intervention group was 152.0 (95% confidence interval [CI]: 147.4–156.5), while the control group mean score was 134.1 (95% CI: 129.1–139.1), p < 0.01. In addition to significantly higher mean total PEACE scores, the intervention group demonstrated better results for each of the seven constructs in the scale (t-test p < 0.05), with the largest differences seen in personal safety, group cohesion and agency. This effect was further elucidated in the FGDs, highlighting the psychosocial benefits of the scholarship programme due to improvements in their academic and financial status. | N/A | 4 app, 1 not app, 3 no info | 27 | 24 |
| [Weinstein et al, 2016](https://centaur.reading.ac.uk/93614/1/Weinstein.%20Enhancing%20need..._removed.pdf) ^98^ | Jordan | Syrian refugees | Needs satisfaction  (Variable, II) | Control group | N/A | 41 | *Purposive sampling*  Participants were recruited by humanitarian aid workers and volunteers of a charity foundation (Mulham Volunteering Team) that had been providing aid for Syrian refugees for nine months prior to the intervention. | Psychological need frustration, depressive symptoms (CES-D), generalised stress (PSS), PTSD symptoms | The one-week long intervention alleviated some of the need frustration likely associated with refugee status, a major aim of the intervention, and lowered refugees’ self-reported symptoms of depression and generalized stress as compared to the comparison condition, though it did not reduce symptoms of PTSD. | 0 | 3 app, 0 non-app, 5 no info | 28 | 22 |
| [Hagen-Zanker et al](https://onlinelibrary.wiley.com/doi/pdf/10.1111/issr.12166), 2018 ^99^ | Jordan | Urban Syrian refugees | UNHCR  Monthly cash transfers  (12, I) | No comparison group | 128,000 (estimate) | 32,000 | *Purposive sampling*  The program is financed through humanitarian funding and delivered by UNHCR, but its targeting method based on vulnerability and the monthly provision of assistance makes it more similar to national social protection programmes, compared to one-off or short-term humanitarian transfers. Applicants who are eligible can apply and transfer payments are determined based on household size and vulnerability level. | Direct effects: access to shelter, health and education, access to employment. Indirect effects: enhanced mental wellbeing, reduced child labour | 48 IDIs and 12 FGDs were conducted with men and women. Of these, 37 IDIs and 7 FGDs were with UNHCR cash transfer beneficiaries, and 10 IDIs and 5 FGDs were with non-beneficiaries. In total, we interviewed more than 140 Syrian refugees, across 60 interviews and group discussions. Almost all beneficiaries used the transfer to pay rent, and that this reduces stress and anxiety among beneficiaries. These effects are important but depend on the continuation of cash transfer support. | 0 | 3 app, 3 not app, 2 no info | 24 | 18 |

# Appendix E: Visual Presentation of the Efficacy-Effectiveness of Included Studies (n= 38)

# Appendix F: Scatterplot showing Quality Rating and Most-to-Least Feasible and Implementable Studies (n= 38).

**
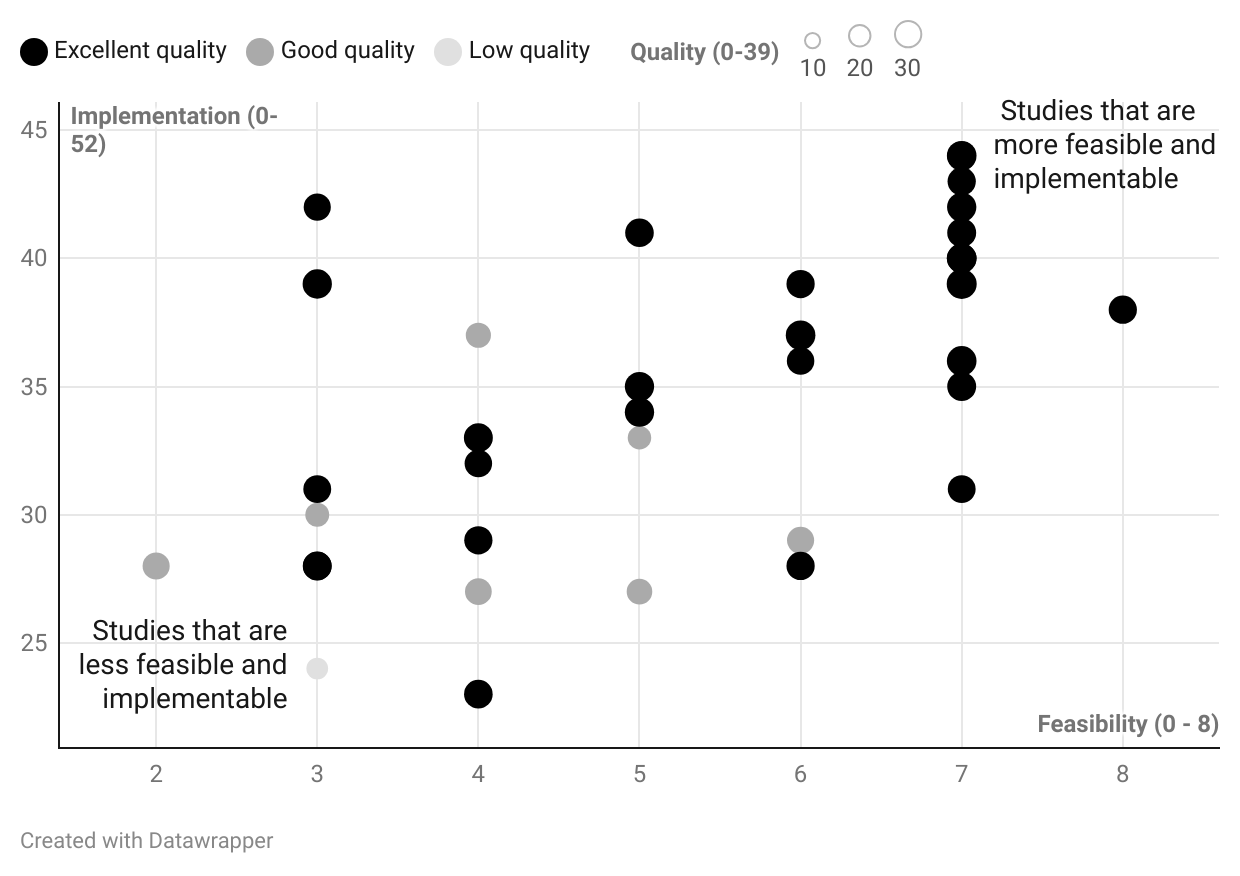
**

Note: Larger, darker circles represent higher quality studies.

# Appendix G: IC-ADAPT Links for top ten studies

|  | **First Author, Year,**  **Intervention** | **Study Design/**  **Intervention Type** | **Population** | **ADAPT** | **Integrative Complexity (IC)** | **IC-ADAPT** |
| --- | --- | --- | --- | --- | --- | --- |
| 1 | Miller et al, 2023  Caregiver Support Intervention (CSI) | Design:  Two-armed RCT (CSI vs. WLC arm)  Type: Parenting skills, group-based | Syrian refugee caregivers  N= 476  Intervention (n= 236),  Control (n= 240) | **Existential Meaning:** Intervention recognised potential cultural discomfort and used relaxation exercises rather than mindfulness; it tackled cultural norms regarding parenting and childhood; and recognised the importance of involving men in mental health interventions.  **Safety/Security:** A broken system due to the instable setting where authors referenced studies examining how “an increase in harsh parenting and a decrease in warm and responsive parenting stemmed from heightened stress due to poverty, inadequate housing, and other environmental stressors.”  **Bonds/Networks:** Group-based intervention providing emotional and practical support for caregivers; targeted child-parent bonds; and created community-based networks to buffer against stress and isolation.  **Justice**: Empowering parents to care for their wellbeing and become better parents, indirectly improving child’s wellbeing; unfair to blame parents for harsh parents when adverse circumstances to some extent prevent parents from applying parenting knowledge.  **Identities/Roles:** Intervention focuses on parenting and father involvement, successfully recruiting and retaining fathers through the intervention by accommodating to their work restrictions. | **No differentiation**: Harsh parenting may indicate compartmentalised thinking, categorical rejection of other viewpoints, single evaluative rule, simple inclusion/ exclusion rules, lack of response differentiation; may indicated that stressors are experienced as sense of threat  **Low differentiation –** Reduced harsh parenting may indicate conditional or emerging recognition of alternate viewpoints, exceptions to the rule, increase tolerance for ambiguity; Improved wellbeing enabled more nuanced handling of stressors enabling more positive interactions with children; Some increase in wellbeing  **High Differentiation –** Further reduced harsh parenting may indicate recognition of multiple alternatives and/or multiple perspectives, validity of different temporal perspectives – I used to use harsh punishment because I was so stressed, now I have a greater sense of wellbeing and I can be more patient with my children, listen to them and try to understand them more, with change attributed to the learning during the intervention; CSI showed significant effects on harsh parenting (d = .17, p < .05; d = .19, p < .05), parenting knowledge (d = .63, p < .001; d = .50, p < .001), and caregiver distress (d = .33, p < .001; d = .23, p < .01). | There were somewhat recognised interactions between ADAPT systems and individual/ group reactions to difference and disagreement. For example, the authors recognised the effects of the current environment (lack of safety/security) on parents' abilities to use existing knowledge to parent well. |
| 2 | El-Khani et al, 2021  Teaching Recovery Techniques (TRT) | *Design*:  Three-armed RCT (TRT, TRT + Parenting, WLC)  *Type*: Multimodal: Trauma-focused CBT + Parenting Skills, group-based | Displaced Syrian families with children aged 9-12 years old  N= 119  TRT + P (n= 41),  TRT (n= 38),  WLC (n= 40) | **Existential meaning:** Mentioned implicitly only in that family integration and dynamics are integral to parental and child wellbeing.  **Safety/Security:** The new parent training sessions included ‘attending to safety' and supported caregivers to plan for the future, including security concerns.  **Bonds/Networks:** The intervention used and recommends an ecological family approach for children and parents rather than an individual approach; improved child-parent relationship and mental health of parents and children; + parent sessions included community building in displacement contexts.  **Justice**: Not mentioned but implicit; empowering parents to care for their kids indirectly improves the family wellbeing.  **Identities/roles:** Integrated with bonds and networks, e.g., + parent sessions included caregiver self-care; noted important role of parents in child's life; no mention of professional/ ethnic/ vocational/ religious roles or identities and recognises that techniques spread through the family. | **No differentiation:** At pre-intervention, parents and children demonstrated high levels of PTSD and anxiety, including avoidance, hyperarousal, and hypervigilance. Lack of response differentiation – one response given to a broad range of stimuli. Possibly conflict avoidance and prescriptive generalisations to stay safe.  **Low Differentiation – recognising a wider range of choices**: Parent sessions taught listening, communication skills, which implicitly supports conditional acceptance or emerging recognition of other perspectives – perhaps exceptions to the rule and increased tolerance of ambiguity. Parents were also taught a range of response choices and strategies to support child's behaviour, struggles; children taught response strategies for memories, nightmares, flashbacks, difficulties in relaxing, concentrating, sleeping, fears.  **High Differentiation – temporal perspectives**: TRT + P group - caregivers reported significant reductions in depression, anxiety, and stress; children in this group showed the greatest levels of improvement in behavioural and emotional difficulties; participants’ earlier behaviour not invalidated, while later behaviour (post-intervention) affirmed.  Temporal perspectives: participants recognised how new perspectives or approaches can grow out of old ones or that earlier and later perspectives cannot simply be dismissed as wrong.  **Integration:**  Implicit in the ecological approach is recognition that the sum of the family unit is bigger than the individual members. Mutual influence and interdependence, negotiation, synthesis. | This was contextualised in terms of recognising the adverse effects of war on families, and the different ways it can affect family members and the family as a unit (bonds/networks and identities/ roles).  It factually recognises how people adapt to cope with adverse experiences and environments in adaptive or maladaptive ways, i.e., Teaching Recovery Techniques implies this. |
| 3 | Eskici et al, 2021  Culturally Adapted Cognitive-Based Therapy (CA-CBT) | *Design*:  Two-armed RCT (CA-CBT, TAU)  *Type:*  CBT, group-based | Syrian refugee women  N= 23  CA-CBT (N = 12), TAU (n= 11) | **Existential meaning:** The authors recognised the importance of how culture shapes people’s treatment expectancies, idioms of distress, experiences, and explanations of mental health and to implement therapeutic interventions that are compatible with the population’s worldview, beliefs, and culture. The intervention was culturally adapted to be culturally sensitive, recognising different expressions of distress, addressing stigmatization, and somatic symptoms along with emotional and cognitive symptoms.  **Safety/Security:** The study recognised the lack of safety/security for refugees due to the chronic exposure to threats and post-trauma.  **Bonds/Networks:** The intervention was in a group format, recognising social relations and women support groups as common coping practices among Syrian women. It was held in a community centre rather than a clinic, which may have encouraged participants’ commitment to treatment and decrease drop-out rates.  **Justice: T**he literature recognised women and children as being more vulnerable to oppression, persecution, and torture.  **Identities/roles:** Syrian refugee women were at a higher risk of child marriage compared with their male peers and Syrian women before the war. | **No differentiation:**  Pre- intervention - There WAS a high prevalence of PTSD, anxiety and depression among Syrian refugee women.  **High differentiation – temporal perspectives:**  Post-intervention – CA-CBT was found to be feasible and acceptable for Syrian refugee women, with significant reductions in anxious-depressive distress and PTSD symptoms. The effect sizes demonstrated that CA-CBT was more effective as compared to TAU in terms of symptom severity change, with nearly medium effect size for the HSCL-25 (d = .40) and very large effect size for HTQ (d = 1.17). | Mental health treatment costs, stigmatization, and the belief that mental health symptoms may get better in time seemed to be the main barriers to seeking treatment among Syrian refugees in Turkey. Related to structural (e.g., language barrier) and attitudinal barriers (e.g., stigma) there is a large mental health treatment gap among Syrian refugees in Istanbul, with a recent study indicating that less than 10% of Syrian refugees with mental health problems seek psychological help. By developing a culturally sensitive intervention that addresses these barriers to accessing MHPSS services in this population (e.g., hosting it in a group format in a non-clinical setting), CA-CBT appears to be feasible and acceptable in this population. |
| 4 | Ponguta et al, 2020  Mother-Child Education Program on Parenting Stress and Disciplinary Practices (MOCEP) | *Design*:  Pilot two-armed RCT (MOCEP, WLC)  *Type:*  Parenting skills, early childhood parenting education, group-based | Mother-child dyads of Palestinian and Syrian refugees, and low SES Lebanese families  N= 106  Intervention (n= 53), WLC (n= 53) | **Existential meaning: N/A**  **Safety/Security:** Implicit to the intervention - 1) Emotional safety: the MOCEP intervention aimed to improve maternal knowledge and disciplinary style with their kids to reduce harsh disciplining. 2) Authors assessed needs satisfaction via household crowding index, human security index (risk of violence), and food security index.  **Bonds/Networks:** MOCEP’S main objective is to improve bonds between mother and child that trickles into the larger family unit; social support is assessed as a secondary outcome.  **Justice:** Maternal empowerment is assessed as a secondary outcome, but this this mechanism is not described in the paper.  **Identities/roles:** implicit to the intervention as the overall aim is to improve the overall wellbeing of the mother-child dyad. The mechanism of change is enhancing parenting style (disciplinary style, parenting knowledge and practice) and reducing parental psychological distress to improve child wellbeing. | **No differentiation:** Pre-intervention, this is implicit as it recognises the prevalence of harsh parenting among the refugee mothers.  **High differentiation:**  Temporal perspectives - post-intervention, mothers showed reduced harsh disciplinary practice and parenting stress.  **Integration**:  Mutual interdependency between mother and child is implicit in the intervention. The focus of the intervention is on improving the wellbeing of the mother and child to enhance family dynamics, thereby recognising the importance of the family unit over the individual. | It is implicit in the intervention that the family (bonds/networks and identities/ roles pillars) is larger than the individual.  Authors comment briefly on not addressing cultural practices of parenting and the poor results for child outcomes but do not do so in-depth: *“Although an assessment of the cultural determinants of parental practices was beyond the scope of this study, we fully acknowledge that culture, parental cognitions, and parenting practices are interrelated.”* |
| 5 | Acarturk et al, 2015  Eye Movement Desensitisation and Reprocessing (EMDR) | *Design*:  Pilot two-armed RCT (EMDR, WLC)  *Type:*  EMDR, individual-based | Syrian refugees  N= 29  Intervention (n= 15), WLC (n= 14) | **Existential meaning:** N/A  **Safety/Security:** The lack of financial security from unemployment and feelings of insecurity for the future are common when living in refugee camps. refugees were exposed to various traumatic events including the death of family members, threatened death to self or others, serious injury to self or loved ones, husbands being at war, and lack of shelter.  **Bonds/Networks: D**isplacement led to the loss of loved ones through death or separation, not being able to bury them, arrested family members, and weakened social network.  **Justice:** The lack of injustice and exposure to human rights abuses were prevalent amongst this population.  **Identities/roles:** N/A | **No differentiation:** with repeated and prolonged traumatization, as well as difficulties with living in exile, are combined with worries about the future, the risk for mental health problems such as depression, anxiety disorders, and posttraumatic stress disorder (PTSD) increases.  **High differentiation**: post-intervention – both PTSD and depression decreased in the intervention group (PTSD: M22.87, SD20.27 vs. M54.21, SD16.26), F(1, 26) 24.166, pB0.001, d1.78, 95% CI (0.92, 2.64); (depression: (M10.15, SD9.60 vs. M20.79, SD7.92) than the wait-list group, t (25)3.15, p0.004, d1.14, 95% CI (0.35, 1.92).  **Synthesis:** Through EMDR, participants integrated traumatic memories via gradual processing of memory into a more positive cognition. | Authors recognised that feasible, cost effective MHPSS interventions are needed, and that cultural stigma was a major barrier to seeking psychological treatment among Syrian refugees, recognising the interaction between cultural beliefs, stigma, and how this leads to maladaptive responses (e.g., manifestation of mental health disorders like anxiety, PTSD, and depression; lack of healthcare seeking). |
| 6 | Ponguta et al, 2019  Mother-Child Education Program on Parenting Stress and Disciplinary Practices (MOCEP) | *Design*:  Implementation Evaluation  *Type:*  Parenting skills, early childhood parenting education, group-based | Mother-child dyads of Palestinian and Syrian refugees, and low SES Lebanese families  N= 106  Intervention (n= 53), WLC (n= 53) | **Existential meaning:** Cultural acceptance of the intervention was emphasised, and the cultural context was assessed to determine barriers and facilitators to program enrolment and adherence.  **Safety/Security:** Mothers and fathers residing in the refugee communities expressed a sense of insecurity and lack of safety. In addition, the community leaders remarked on the increase in the influx of Syrian refugees, which they felt had increased inter-ethnic tensions among the communities; economic need and unemployment, as well as a sense of loss and hopelessness, emerged as common factors. These, in turn, affected the perceptions of the impact that the environment had on children’s wellbeing, which included concerns around safety.  **Bonds/Networks: P**re-intervention - an overarching sense of mistrust, lack of unity, and challenges to socializing with the larger community. Post-intervention – implicit to intervention that enhancing family dynamics and bonds (especially mother-child dyad) was the main goal.  **Justice: I**mplicit to intervention by empowering mothers. Authors emphasised the need to characterise the context within the intervention to “*determine ways in which existing practices, beliefs, and talents of the mothers can be included to sustainably empower the communities, while working in partnership with the mothers and families themselves to recognize their own potential and capita*l.”  **Identities/roles:** One of the major barriers to enrolment was the perception that parenting knowledge is innate and cannot be learned or developed. As one mother recalled, quoting her friend: *“Why do you need to go to the Mother and Child Program? Why don’t you know how to raise your children?”* | **No differentiation:** Pre-intervention, this is implicit as it recognises the prevalence of harsh parenting.  **Low differentiation:**  Enablers to joining MOCEP program included 1) openness and interest in new content and acceptability of exploring parenting strategies: “*People who are close to me encouraged me and said it is a good idea. Even if we are educated there are still things and skills that we don’t know so it is good to learn them from someone with experience*.” 2) Mothers expressed interest in learning new approaches to parenting—with an emphasis on effective strategies to discipline their children—as well as in promoting their own and their children’s social skills. Mothers were introduced to wider ranges of choices to disciplining.  **Integration:**  Post-intervention benefits were greatest among mothers who attended >14 sessions - disciplinary practices improved and were largely focused around listening, dialogue, and redirecting behaviour. Corporal punishment and yelling were reported at endpoint to be used less frequently, compared to frequencies reported at baseline. Mothers also reported changes in their own social and communication skills, as well as in their own feelings of resilience (e.g., ability to cope with stress) and wellbeing. Regarding perceived changes in the child, several mothers reported increased confidence in their children. | Authors acknowledged the role of the environment (structures/ systems) in influencing [harsh] parenting, mother-father relationship and wider community relationships (e.g., arrival of more refugees) and targeted parenting knowledge and practices, and maternal empowerment as mechanisms of change to enhance family dynamics, well-being, and that of the greater community. |
| 7 | Bryant et al, 2022  Group Problem Management Plus (gPM+) | *Design*:  Two-armed RCT (intervention, EUC)  *Type*:  Multimodal = Teaching skills in arousal reduction, problem-solving, behavioural activation, and accessing social support; group-based | Camp-based Syrian refugees (male and female)  N= 410  gPM+ (n= 204) and Enhanced Usual Care (EUC; n= 206) | **Existential meaning: N/A**  **Safety/Security:** implicit through assessing parenting behaviour and its impact on children's self-reported mental health  **Bonds/Networks:** gPM+ intervention Session 4 teaches participants how to access social support (increase bonds/networks); the intervention is conducted in group format to encourage building new connections.  **Justice: N/A**  **Identities/roles: N/A** | **High differentiation – temporal perspectives:** post-intervention, there was a significant reduction in depression, disability, and disciplinary parenting behaviour. Reduced inconsistent disciplinary parenting (e.g., hitting, shouting) indicates change over time with understanding that depression and anxiety can increase harsh parenting and reduced depression and anxiety can reduce harsh parenting.  **Integration:**  Reduced harsh parenting associated with reduced attentional and internalising problems in refugees’ children; reducing maladaptive behaviour can have positive impacts on children's mental health. | Some of the taught strategies may be limited in their application in the camp context because of restricted movement within the camp, limited opportunities to gain employment, constraints on making decisions that impact one's future. Separation from family after displacement can compound psychological difficulties and hamper the extent to which problem management strategies may be employed. Ongoing stressors in the camp may have mitigated potential gains from gPM+. |
| 8 | Acarturk et al, 2022  Group Problem Management Plus (gPM+) | *Design*:  Pilot two-armed RCT (intervention + ECAU, ECAU only)  *Type*:  Multimodal = Teaching skills in arousal reduction, problem-solving, behavioural activation, and accessing social support; group-based | Syrian refugees (male and female)  N= 46  gPM+ and Enhanced care as usual (E‐CAU) (*n* = 24) or E‐CAU only (*n* = 22). | **Existential meaning:** Adapted gPM+ for Syrian refugees in Turkey; inclusion/exclusion criteria adapted for Turkey; all materials linguistically and culturally adapted to make suitable for Syrian population (no details)  **Safety/Security:** The current context for Syrian refugees after displacement and arriving to their host countries is unsafe/unsecure, where they have experienced various post-displacement problems such as discrimination, economic problems, and language barriers.  **Bonds/Networks:** The group format of intervention (Group PM+) and non-specialist Arabic speaking facilitators who are peer refugees allows for building trust and new connections.  **Justice: N/A**  **Identities/roles: N/A** | **No differentiation:** Some participants reported that listening to other's problems induced stress (freeze/flight)  **Low differentiation:**  Perceived benefits of gPM+ were the opportunity to share problems and concerns with each other in a group | The authors acknowledged the possible effects of current events on participant mental health, e.g., operation peace spring launched in northern Syria at the border with Mardin, causing fears of deportation, possibly increasing risk for mental disorders, and first COVID lockdown which cut off core services and income generating activities for refugees, causing severe economic hardships.  gPm+ should be applied in tandem with strong advocacy for the protection of those who face adversity and for services that address social, physical and broad mental health needs. |
| 9 | Sieverding et al, 2022  AMENAH early marriage intervention | *Design*:  Pilot cohort (pre-post) mixed methods  *Type:*  Community-based participatory, group-based | Syrian refugee girls aged 11–14 years who are at high risk of school dropout and early marriage, and their mothers  N= 203 schoolgirls and their households | **Existential meaning:** Cultural workers rather than outsiders delivered the intervention because they had knowledge of their community - but they experienced the same social/ economic/ legal hardships as the participants; the researchers are based at a local university and have made cultural knowledge informed decisions as well as utilised scientific evidence. The empowerment-based approach is limited when structural, legal and economic constraints on refugees cannot be addressed. For example, although Lebanon committed to including Syrian refugee children in the public education system, adopting a condensed afternoon shift for them, the official education policy for Syrian refugees was not uniformly implemented and full integration into formal schools has faced numerous challenges.  **Safety/Security:** Early marriage is perceived as a form of (economic, social, psychological) protection from harassment and sexual violence in the community. Yet it has more harmful consequences including school dropout, limited access to healthcare, and increased maternal mortality and poor reproductive health outcomes. The intervention utilised a ‘safe spaces’ approach: it was held amongst peers in girl-only spaces, and used a well-known and respected, accessible NGO premises; families often prioritise (physical) security over education despite preferences for education.  **Bonds/Networks:** Part of the intervention design was to set the intervention among peer networks and support peer relationships; peer networks were seen as important for programme attachment and possibly content acceptance, noting that displacement can disrupt peer networks and lead to social isolation.  **Justice:** Implicit to the aim of the study, where researchers aimed to enable and increase opportunities to pursue education rather than marriage alone through school retention and girls’ right to education. Cultural workers (facilitators) were paid as a matter of justice as they are also facing legal/ economic/ social hardships.  **Identities/roles:** Intervention activities stressed skill building and were designed with a focus on gender equality, encouraging girls to examine their perceptions. Cultural workers were trained on their roles and responsibilities from gender, familial, cultural and ethnic identities. | **No differentiation:**  Fear of insecurity (physical/financial/ psychological) leads to early marriage.  **Low differentiation**: Attendance of sessions reflected acceptance of the intervention, where mothers’ attendance was considerably more variable than that of girls. The intervention aimed to teach a wider range of response choices to fear of insecurity (e.g., continued education and opportunities) rather than just early marriage.  **High differentiation – temporal perspectives:**  Girls thought they had to marry early and now realised they don’t. Mothers reported increased openness, confidence, maturity, and ability to defend themselves in their daughters.  **Integration – mutual dependency:**  Intervention designers realised they needed more information to retain father's involvement and increase mother's involvement. Mothers perceived the positive character changes in their daughters to have improved their relationship with each other.  **Integration – need more information/ sum is bigger than its parts:**  Empowerment is limited when structural, legal and economic constraints on refugee households cannot be addressed | The authors recognise the interactions between structures/ systems and individual/ group reactions to training/ child marriage/ education and have contextualised it through their community-based approach by involving cultural workers and refugees in the co-design and implementation of the intervention to reduce the barriers to feasibility and acceptance of the intervention.  Without an integrated approach that addresses structures and systems, there will be some maladaptive reactions (e.g., child marriage) that are rooted in and likely to increase due to social norms and family honour. |
| 10 | Bunn et al., 2022  Sharing Stories Eases Pain – Group Intervention | *Design*:  Qualitative cohort (post only) methods  *Type:*  Counselling + Physiotherapy (multimodal), group-based | Syrian refugees  N= 31  No comparison group | **Existential meaning:** Collectivist cultures are defined by strong social relationships, reflecting values and mirroring traditional help-seeking approaches exchanged between members; the intervention was found to be culturally acceptable and addresses social-relational losses.  **Safety/Security:** Post migration, physical safety exists for refugees but financial and emotional safety does not - refugees face struggles to build community and livelihood in a host country and deal with the erosion of capacity to trust.  **Bonds/Networks:** Disrupted attachments from forced migration, dislocation, grief; breakdown of social networks paradoxical to cultural norm of interdependence, contribute to isolation.  **Justice:** The intervention aimed to empower participants to learn to respond and support others to ease their pain when sharing stories - becoming in control of their experiences rather than victims.  **Identities/roles:** Refugees often describe a sense of social dislocation and grief resulting from the loss of meaningful roles and connection to a community. | **No differentiation:**  Pre migration, the instability led to fear, mistrust, polluting community ties. During the intervention, for a small group of participants, rather than galvanize relationships, the experience was overwhelming and led to feeling insecure and uncomfortable around group members.  **Low differentiation:**  Participants learned a wider range of responses choices regarding relationships.  **High differentiation:** there was a recognition and temporal changes in participants, including forming caring relationships, alleviating pain through physiotherapy, and Syrian men and women used the term “relief” to capture the cathartic quality associated with the open expression of strong feelings and experiences.  **Integration:**  Recognition of mutual interdependence in that sharing stories with others eases pain. Withholding judgment on pain and synthesis of trauma: Participants made sense of the pain in the storytelling process as a necessary condition toward relief, invoking the metaphor of a poorly treated wound that needs to be reopened and cleaned to heal. | The authors recognise interactions between broken/ disrupted structures/ systems (political terror, war, forced migration) and adaptive/ maladaptive reactions among individuals/ groups including isolation and lack of trust.  The intervention focused on enhancing bonds/networks and integrating and synthesising traumatic experiences, as well as encouraging an adaptive response to pain by sharing stories. |

# Appendix H: IC-ADAPT synthesis of top ten studies

| **Reviewer** | First  Author | Publication Year | **Integrative Complexity (IC)** | **IC component: Differentiation** | | | **IC component: Integration** | | | **IC-ADAPT** | **links between ADAPT systems and IC variables** |
| --- | --- | --- | --- | --- | --- | --- | --- | --- | --- | --- | --- |
|  |  |  | **No differentiation, no integration** | **IC scores 2 and 3** | **Not black and white thinking but knowing options vary:** | | **IC scores 4, 5 and above** | | **The family the community is bigger than the son the mother etc** | **Will be there to a greater to a lesser extent - some question the injustice more than others. Not all give full or detailed context. So just copy and paste from the article / paste as you find** | *Responses should be captured from authors perspective: Example - not specified but implicit through xyz* |
|  |  |  | *Fight (physical or verbal argument), flight (leave, run or back away), freeze (or flock: go limp, do nothing), fawn (comply or people please), flock responses (feeling the need to be with others) can be what is required in the situation* | *Increased tolerance for ambiguity; probability statements; conditional acceptance; exceptions to the rule; acceptance/ tolerance of other viewpoints as relevant, legitimate, justifiable, valid even if disagree - may create a tension* | *Recognising a wider range of response choices yet not seeing any relationship among the response choices* | *Temporal perspectives - change over time without dismissing what used to do (e.g., I used to do this, now I do that), with change attributed to the learning during the intervention* | *A tension indicating a mutual dependency; if they do something different maybe the other will do something different* | *withholding judgement - need more information; relationship represented by a tension between alternatives; a superordinate statement that integrates alternatives/ viewpoints.* | *Alternatives are to some degree legitimate and can be combined that is more than the sum of the parts; negotiation; synthesis; causal attribution.* | *Contextualises the conflict/ opposition/ tension by recognising the interaction between structures/ systems and how individuals/ groups react to difference and disagreement.* | *Interactions between environment and individuals/ groups; effects of varying levels of stress arousal; adaptive and maladaptive responses/ reactions* |
| **LB** | [Bryant et al](https://journals.plos.org/plosmedicine/article?id=10.1371/journal.pmed.1003949) | 2022 | Prolonged grief (PG-13; assesses if grief causes significant functional impairment = FREEZE?) assessed showed no improvements after intervention. | no info | no info | Quant results showed (intervention arm had) a greater reduction in depression, PSYCHLOPS ie disability (main problems experienced, functioning, and well-being), and disciplinary parenting behaviour (APQ inconsistent discipline subscale) than control group after 3 months follow-up. | no info | no info | no info | Group-based programs may be more cost-effective than individually administered psychological interventions [57], and when they can be effectively delivered by nonspecialists, this further increases the likelihood that programs such as gPM+ could be implemented in refugee camps in poorly resourced countries [58]. Further, group interventions can be more acceptable in collectivist societies, including Syrian refugees | Intervention not as effective as other similar trials because: 1) post-migration challenges (moving to a camp, leaving Syria and loved ones, loneliness), 2) Several participants had PTSD/exposed to trauma constantly and a brief intervention may not be effective for them. 3) Follow-up assessment happened during COVID which could have impacted the true effect of the intervention |
| **EB** |  |  | no info | no info | reduced inconsistent disciplinary parenting (hitting, shouting) implies other response choices were evidence | reduced inconsistent disciplinary parenting indicates change over time with understanding that depression and anxiety can increase harsh parenting and reduced depression, and anxiety can reduce harsh parenting | reduced harsh parenting associated with reduced attentional and internalising problems in refugees’ children; reducing maladaptive behaviour can have positive impacts on children's mental health | no info | no info | some of the taught strategies may be limited in the camp context because of restricted movement within the camp, ltd opportunities to gain employment, constraints on making decisions that impact one's future; refugees in camps may have restricted movement, limited employment opportunities, and separation from family that can compound psychological difficulties and hamper the extent to which problem management strategies may be employed | ongoing stressors in the camp may have mitigated potential gains from gPM+ |
| **LB** | [Acarturk et al](https://link.springer.com/article/10.1186/s12888-021-03645-w) | 2022 | Some participants reported that listening to other's problems induced stress (freeze/flight??) and another participant stopped attending because their problems were perceived as not as important (flight) | Perceived benefits of gPM+ were the opportunity to share problems and concerns with each other in a group | no info | no info | no info | no info | no info | 1) No significant differences in health system costs or in productivity losses between the two groups were found. 2) It is also important to recognise the complexity of the health system in Turkey, even after major reforms which have consolidated several insurance funds into one public insurance system and ensured free of point access to primary care | The intervention was found to be acceptable and feasible, given the potential costs to productivity, accessibility, cultural sensitivity of delivering it for Syrian refugees in Turkey. |
| **EB** |  |  | no info | no info | no info | no info | no info | no info | no info | target population group experience a wide range of post-displacement problems such as language problems, discrimination, economic problems; do not access mental health care due to language problems, stigma, lack of culturally adapted psychosocial interventions | providing adapted intervention delivered by peers in group settings may have addressed interactions between structures/ systems and individual / group reactions |
| **LB** | [El-Khani et al](https://www.proquest.com/docview/2565252766?pq-origsite=gscholar&fromopenview=true) | 2021 | Fight + Freeze + Flight + Flock: Children and parents both assessed for trauma/PTSD (CRIES + IES-R) and anxiety (SCARED + DASS) showed high levels pre-intervention. Ex: IES-R subscales avoidance (Freeze and Flight) / hyperarousal including hypervigilance (Flock). Parents might show anger (IES-R intrusion, parenting competence) | no info | no info | Quant results: children in the enhanced TRT + P group showing the greatest levels of improvement in behavioural and emotional difficulties compared to children in the TRT or waitlist control groups. Caregivers in the TRT + P group also reported significant reductions in depression, anxiety, and stress. | no info | no info | Higher parental competence, self-regulation, wellbeing can lead to stronger family unit | The findings showed the value of working with the family as a unit (BONDS/NETWORKS + IC scores 2-3 temporal perspectives), rather than as individuals, supporting an ecologically based view which has been increasingly expressed by many authors [27,28]. The family provides the enduring context in which children grow and develop; enhancing the skills of caregivers to cope with novel challenges and changes in their children’s behaviours and mental health can have an important preventive effect. The enhanced intervention, in addition to improving child mental health, showed the capacity to produce significant improvements in two of the key predictive factors identified by a major review by Scharpf and colleagues [28]: parental mental health and impaired parenting. These findings support research that acknowledges that parents experience psychosocial benefits from parent training interventions even without explicit attention to their own wellbeing [58]. Enhancing the capacity and effectiveness of the family to nurture the child is crucial in low-resource contexts where provision of mental healthcare and support may be extremely limited. | Understanding the ecosystem of family/parent/child wellbeing = The enhanced intervention, in addition to improving child mental health, showed the capacity to produce significant improvements in two of the key predictive factors identified by a major review by Scharpf and colleagues [28]: parental mental health and impaired parenting. |
| **EB** |  |  | implicit in terms recognising mood changes, creating safety/ security but not explicit | parent sessions taught listening, communication skills, which implicitly supports tolerance of other viewpoints | parents taught a range of response choices and strategies to support child's behaviour, struggles; children taught response strategies for memories, nightmares, flashbacks, difficulties in relaxing, concentrating, sleeping, fears | change over time plotted; earlier behaviour not invalidated while later behaviour affirmed. | approach of programme recognises interdependency and mutual influence of parents/ caregivers and children in an ecological approach | withholding judgement and getting more info may be one of the skills embedded in parenting sessions - ask questions | implicit in the ecological approach is recognition that the sum of the family unit is bigger than the individual members | *contextualised in terms of recognising adverse effects of war on families, and the different ways it can affect family members and the family as a whole (bonds/networks and identities/ roles pillars)* | factually recognises how people adapt to cope to adverse experiences and environments in adaptive or maladaptive ways, i.e., Teaching Recovery Techniques implies this. |
| **LB** | [Ponguta et al](https://www.sciencedirect.com/science/article/pii/S0890856720300666) | 2020 | **Fight**: at baseline, mother assessed for human security index (exposure to violence) and parenting disciplinary style with children. **Flock**: exploratory outcome looks at dyadic interaction between mother and child and whether child goes to mother (not significant outcome in intervention group) | no info | no info | Maternal outcomes: mothers in intervention arm showed reduced harsh disciplinary practice (primary) and parenting stress (Secondary) | no info | no info | The intervention focuses on the family unit to ensure the wellbeing of individual family members: MOCEP is a group-based program designed to foster positive parenting practices and to promote early childhood development. | Some reflections on not addressing cultural practices of parenting and poor results for child outcomes but not in-depth: Although an assessment of the cultural determinants of parental practices was beyond the scope of this study, we fully acknowledge that culture, parental cognitions, and parenting practices are interrelated.6,41 We also acknowledge that relying on questionnaire data to assess parental practices has limitations in cross-cultural parenting research. | Our study demonstrated that mothers who participated in MOCEP reported less harsh disciplinary practices. Parenting programs have shown a positive impact on stress arousal and maladaptive reactions (disciplinary style on children) but this was only shown in high-income countries. This is the first study to show it in LMICs, though the results are not significant for child outcomes with exception to ALEF pragmatic scores. This is explained by children already having access to early childhood development and education programs. |
| **EB** |  |  | implicit to harsh parenting | could be implicit to outcome but not enuf info | could be implicit to outcome but not enuf info | could be implicit to outcome - used to do this now do that | implicit in intervention - parent-child mutual influence | could be part of changes in parenting style | | *poverty, war, displacement can drive family instability, compromising parental well-being and positive parenting practices, with detrimental effects on child-development* | implicit in underlying rationale for intervention, but unclear if considered in intervention |
| **LB** | [Acarturk et al](https://www.tandfonline.com/doi/pdf/10.3402/ejpt.v6.27414) | 2015 | **Fight/Flight/Freeze:** When repeated and prolonged traumatization, as well as difficulties with living in exile, are combined with worries about the future, the risk for mental health problems such as depression, anxiety disorders, and posttraumatic stress disorder (PTSD) increases. | No info | No info | **1) PTSD lower in intervention group post-intervention and this effect remained in follow up:** the EMDR group had significantly lower IES-R scores at posttreatment as compared with the wait-list group (M22.87, SD20.27 vs. M54.21, SD16.26), F(1, 26)24.166, pB0.001, d1.78, 95% CI (0.92, 2.64). Between the immediate post-test and the follow-up, the EMDR group’s trauma scores did not change. **2) Depression lower in intervention group after intervention:** after treatment, the EMDR group had lower depression scores (M10.15, SD9.60 vs. M20.79, SD7.92) than the wait-list group, t (25)3.15, p0.004, d1.14, 95% CI (0.35, 1.92). | No info | No info | Synthesis: integration of traumatic memories via gradual processing of memory into a more positive cognition | **Cultural stigma as a major barrier to seeking psychological treatment among Syrian refugees (interaction between 'existential meaning' = stigma, AND how refugees are likely to seek healthcare):** The present study reports promising results for EMDR as feasible, acceptable, and effective intervention in reducing PTSD and depression symptoms among refugees in a camp setting. However, to conduct an intervention in a refugee camp at the border was not easy for many reasons. As stated, some refugees were afraid of becoming insane because of psychological treatment. This could be partially explained by a low familiarity with mental help in Syria. A previous study about the mental health service use in Arab countries indeed indicated that in Syria there were fewer than 0.5 psychiatrists and no psychologists per 100,000 population in 2007 (IASC, 2007). | **Maladaptive responses (increased MH problems) among refugees due to displacement and need for MH interventions that are feasible and cost effective:** Refugees have higher risk for mental health problems not only compared to host populations but also compared to other migrant groups (Bhugra et al., 2011). Moreover, given the increase in the number of refugees worldwide, it is important to conduct effective interventions both to reduce individual suffering and to prevent future conflicts in the communities. |
| **EB** |  |  | mental health care / support provision in refugee camps vs elsewhere; testing of interventions in camps vs elsewhere | | adaptation of RCT to refugee camp | | relationship between findings with seeking treatment population and non-treatment seeking population - difference acknowledged but further study suggested with economic evaluation |  |  | Besides the past traumatic events, refugees may also have worries about their future. A recent study of refugee psychiatric outpatients in Norway indicated that postmigration stressors such as unemployment, poor social integration, and weak social network are related to mental health problems (Teodorescu et al., 2012). | |
| **LB** | [Miller et al](https://acamh.onlinelibrary.wiley.com/doi/pdfdirect/10.1111/jcpp.13668) | 2023 | **Fight/Flight/Freeze:** caregiver stress and distress stemming from armed conflict and displacement negatively impact parenting through two primary pathways: an increase in harsh parenting and a decrease in parental warmth and responsiveness, which in turn predicts emotional and behavioural problems in children. | no info | no info | **1) Primary outcome = Effect of CSI:** the effect of the CSI at follow-up was larger, and statistically significant in Wave 1 (those who received the full intervention) and non-significant in Wave 2. **2) Secondary outcomes:** Harsh Parenting showed a significant effect at endline as well as follow-up in the full sample. In Wave 1, this effect was only significant at endline. Parenting Knowledge also showed a significant effect both at endline and follow-up, in the full sample and in both Waves separately. Parental Warmth and Responsiveness did not show significant changes. **3) Mediator outcomes =** Caregiver Distress showed a significant effect at both endline and follow-up in the full sample, as it did in Wave 1, but only at endline in Wave 2. The remaining outcomes Psychosocial Wellbeing, Stress, and Stress Management did not show significant changes. **4) Two mediators emerged from this two-step procedure who were independent from each other: Caregiver Distress (p < .001) and Caregiver Psychosocial Wellbeing (p = 0.084).** The proportion of the effect between the CSI and Harsh Parenting mediated by Caregiver Distress was 29% and the proportion mediated by Caregiver Psychosocial Wellbeing was 7.8%. | **Parents became warmer with kids after intervention (Focus group results):** In a separate paper based on focus group discussions with CSI participants from this trial, caregivers spoke consistently about how their improved wellbeing (i.e., feeling less distressed and more relaxed) allowed them to interact more warmly with their children, and to make greater use of positive, non-violent behaviour management strategies—including those they already knew and others learned during the intervention (Miller et al., unpublished data). | no info | Recruiting both male and female parents for the intervention and adhering throughout | **1)** The marked difference in findings between our pilot RCT and this trial **(focused on ADAPT System identities/roles of parenting X Caregiver wellbeing, emotional regulation, stress management, mindfulness, anger management, parenting skills -- High IC)** underscores that as adversity increases **(i.e. ADAPT systems bonds/networks + justice + existential meaning),** so too does the importance of coupling psychosocial programming with (IASC) interventions that address basic needs**:** First, this study was conducted in a context of multiple and extreme adversities, including an economic crisis that has driven Syrian refugees further into poverty; political unrest that evoked distressing memories of war, and a fear among Syrians of becoming caught up in another violent conflict; and the outbreak of the COVID-19 pandemic, followed by a lockdown that stopped all field activities and led to half our sample receiving only part of the intervention. Stress levels were extremely high throughout the trial, and most families were confined in crowded, substandard living conditions for extended periods of time. Such conditions may explain why our effects were modest, and why the CSI did not significantly improve overall parenting, reduce stress or improve caregivers’ capacity for stress management. Pilot which wasn't held in these adverse conditions showed medium to large effect size in comparison. 2) It seems likely that in this trial, we encountered the limits of a stand-alone preventive psychosocial intervention in a context of extreme adversity. Our focus on stress management may simply have been inadequate in the face of the intense stressors that impacted participants throughout the trial. In conditions of extreme adversity, psychosocial interventions should clearly be coupled with other types of support that address basic needs, from nutrition and housing to economic support and healthcare, as well as clinical interventions for severely distressed individuals—a key point made by the IASC in its guidelines for mental health and psychosocial support in humanitarian settings. | **Group 1 receiving both wellbeing and parenting skills saw reduction in stress and harsh parenting, whereas group 2 that only received wellbeing skills, only saw reduction in distress (intervention X adaptive vs maladaptive responses):** When we analysed our findings separately by wave, we found that Wave 1 participants, who received the full intervention, showed lower psychological distress, improved overall parenting, and reduced harsh parenting. In contrast, Wave 2 participants received all the wellbeing sessions but only half of the parenting sessions; correspondingly, they showed a significant reduction in distress, but no change in parenting, or in harsh parenting specifically. Improved wellbeing appears to free caregivers up to parent more effectively, but there is a clear benefit to also learning methods of positive discipline and strategies for increasing positive parent–child interactions. |
| **EB** |  |  | harsh parenting implies fight reactions | focus group findings reported separately or not reported yet - examples mentioned in this article - improved wellbeing enabled more positive interactions with children | N/A | N/A | N/A | N/A | N/A | contextualised; somewhat recognised interactions between structures/ systems and individual/ group reactions to difference and disagreement | was recognition of the effects of the situation on parents' abilities to use existing knowledge to parent well |
| **LB** | [Sieverding et al](https://www.ghspjournal.org/content/ghsp/10/1/e2100079.full.pdf) | 2022 | Flight/Freeze/Fawn: girls were dropping out early from school for early marriage assuming better, safer lives, but in reality, early marriage is associated with school dropout, limited access to reproductive health services including contraception, and early pregnancy, which increases the risk of maternal mortality and other poor reproductive health outcomes.11 Fawn: complying with social norms of early marriage | Attendance of sessions reflected acceptance of the intervention: Mothers’ attendance was considerably more variable than that of girls. Of the 175 mothers in the panel data, 67% attended any session and, among these, the average number of sessions attended was 3 of a total of 8. The attendance outcome for mothers was therefore categorized as attending at least 1 session versus not attending any sessions. Among girls, by contrast, 80% attended 10 or more sessions of a total of 16. | No info | 1) Mothers reporting on changes in daughters: "[My daughter] became more open and talks to me more. As for me, I became more able to understand her. I mean she used to be shy, but now she became courageous." [...] My daughter became more mature. [In the past,] if she was catcalled on the street, she used to cry. Now she became more mature and able to defend herself. And her character became stronger. | Mutual interdependence: Mothers also stated that their daughters became more open with them after participating in Amenah and gained self-confidence and assertiveness, highlighting that these changes improved their relationships with their daughters. | 1) A superordinate statement that integrates alternatives/ viewpoints: Mothers also stated that their daughters became more open with them after participating in Amenah and gained self-confidence and assertiveness, highlighting that these changes improved their relationships with their daughters. | No info | 1) *Community interventions + involving refugees in design/delivery of interventions is key to success* (**trust, building rapport, bonds/networks, providing new identities/roles, empowering them - justice - by contributing to helping community with financial security**) = The Amenah pilot experience highlights the importance of involving refugees in the design and delivery of interventions that serve their community. The female Syrian CWs who implemented Amenah served as a link between the community and the research team and established a trusting relationship with the girls’ parents. The involvement of members of marginalized communities in public health research enhances its relevance and contributes to reducing social inequities.31 2) The importance of involving refugees in delivering health services in humanitarian settings has also been noted in other contexts.32 In displacement settings, however, researchers ought to recognize how economic and legal factors affect implementers from excluded communities who genuinely commit to the research project but whose contributions are constrained by multiple and competing demands. The Syrian CWs who implemented the Amenah intervention shared the same socio-economic background and displacement experience as the girls’ families. While this affinity to the community enhanced cultural understanding and parental acceptance, it meant that CWs faced the same social, economic, and legal hardships that all Syrian refugees in Lebanon face and, as women, endured patriarchy in their own community. **3) Underlying mechanisms of change within the intervention were less understood by the mothers:** Mothers’ focus on the English support sessions is also likely a reflection of the structural barriers that refugee children face in the Lebanese school system. While providing English lessons was important both for families’ commitment to the intervention and for directly supporting girls’ school retention, families’ focus on academic support may undermine their understanding of the components of the intervention that aim to empower girls or address norms around early marriage. 4) These findings again point to the role of structural constraints in shaping the educational outcomes of Syrian refugee girls. **The substantial shortcomings of Lebanon’s education policy for refugees,5,7 along with poverty and insecurity, lead families to prioritize girls’ security over their education despite preferences for the latter.12,14** | 1) involving refugees in design/implementation unlike international investigators who are unfamiliar with the social and political context of the Arab region, raising questions not only about the validity of the findings but also about ethics and power.33 2) involvement of mothers was weak and fathers non-existent due to existing environmental constraints: We were ultimately unable to engage fathers in the intervention, which is likely related to competing economic priorities among this displaced population as well as the perception that matters related to daughters are more the mother’s domain. Attendance among mothers was more variable and appeared to be affected by household-level factors. Although childcare services were provided in mothers’ sessions, transportation or transportation fees were not, which may have reduced the likelihood of attendance. Other researchers have also highlighted how structural inequalities that affect participation stymied the impact of community-based research in the highly inequitable context of Lebanon. |
| **EB** |  |  | fear of insecurity leads to early marriage reaction | perhaps exceptions to the rule, others' viewpoints about education are relevant | hopefully wider range of response choices to fear of insecurity | temporal perspectives - thought had to marry now realise don't | intervention designers realised they needed more information to retain father's involvement and increase mother's involvement | need more information: empowerment limited when cannot address structural, legal and economic constraints on refugee households | sum bigger than parts: empowerment limited when cannot address structural, legal and economic constraints on refugee households | contextualised; recognised interactions between structures/ systems and individual/ group reactions to training/ child marriage/ education | without integrated approach that addresses structures and systems there will be some maladaptive reactions (child marriage) |
| **LB** | [Bunn et al](https://www.tandfonline.com/doi/pdf/10.1080/01933922.2021.2000084) | 2022 | **1) pre migration - Fight/Flight/Freeze:** pre migration, the instability led to fear, mistrust, polluting community ties. **2) during intervention:** for a small group of participants, rather than galvanize relationships, the experience was overwhelming and led to feeling insecure and uncomfortable around group members. | | | 1) **Forming caring relationships** led to a range of other psychosocial benefits described as easing pain, including emotional relief and a sense of belonging or feeling “at home.” **2) Process of sharing stories in the group transformed the meaning** that Syrian men and women attributed **to their narratives**, allowing them to gain new perspectives on their own lives and generating a sense of hope. **3) Alleviating pain through physiotherapy and making connections:** While sharing emotional pain facilitated relationships in the group counselling, sharing physical pain was also an important aspect of social connection in the physiotherapy group. Group members participated in exercises and learned new techniques for alleviating their pain and this exchange of physical pain facilitated closeness between group members. The exchange of stories also went beyond the confines of the group sessions and was fluid in nature – often beginning before sessions started while gathering at the organization, continuing during the group sessions and transitioning to digital platforms outside the sessions, most commonly WhatsApp. **4) Before/after: from suffocated to cathartic relief:** Syrian men and women used the term “relief” to capture the cathartic quality associated with the open expression of strong feelings and experiences. Whereas men and women felt suffocated before, being able to share with group members was described as feeling unburdened, taking things out of your heart, letting things bothering you out and getting things off one’s chest. **5) Sense of belonging and alternate experience**: Syrian refugees in Jordan endured ongoing threats to identity, status, and daily survival based on their refugee status. The urban context was unfamiliar for many and often furthered their sense of alienation and dislocation. The group, however, offered an alternative experience – the protected space of the group recreated aspects of home, where they were treated with care and dignity by providers and other group members. **6) Gaining perspective and sense of hope:** Another dimension of easing pain was the way in which men and women gained perspectives on their lives in ways that led to a sense of hope. Witnessing others who were persevering in spite of similar or worse experiences inspired a sense of strength for their own lives. Through a “river of life” exercise conducted in one of the sessions, group members symbolize their past, present and future as a river, which encounters obstacles but continues to flow. Through this exercise, they were exposed to others with similar experiences and together, they began to envision a future in ways they had not done since arriving in Jordan. | 1) Mutual interdependence: a) Sharing stories eases pain = “Seeing other’s problems eases yours,” which participants frequently used to describe experiences and benefits of sharing lived experiences with other group members. b) separated from relationships at home, these friendships played a significant role in men and women’s lives. Ramiz, a 54-year-old man described this as something small but big at the same time. He said, “when you feel that somebody cares about you, it’s something great.” | Withholding judgment on pain: Participants made sense of the pain in the storytelling process as a necessary condition toward relief, invoking the metaphor of a poorly treated wound that needs to be reopened and cleaned in order to heal. | no info | 1) The group-based intervention mechanism of change is to create group relationships (**bonds/networks)** and interpersonal learning opportunities **(existential meaning and perhaps justice?)** to affect positive outcomes [to address the inadequate structures/systems addressed in point #2p.. 2) Recent research conducted with Syrian refugees in Jordan identified the **breakdown of social networks** in ways that are paradoxical to the cultural norm of interdependence and contribute to experiences of isolation (Stevens, 2016). These particular losses and traumas are inevitably exacerbated by the material and resource problems that mark life as a refugee – poor access to food and health care, ambiguity about one’s status and future, and ongoing struggles for daily survival, discrimination, among other problems | **1) Enhancing bonds/networks enhanced a sense of belonging (existential meaning) and integration/synthesis of traumatic experiences such that meaning making became a positive experience:** In this study we learn that gathering with those of similar age and gender reflected the religious and cultural context in Syria and contributed to a sense of comfort and familiarity in the groups. While participants joined groups that were homogeneous and heterogenous by nationality, participants emphasized that shared life experience, more so than nationality, provided the primary basis for relating and connecting. **2) bonds/networks improve --> enhances social support + adaptive response:** Acknowledging aspects of their own experiences fostered support from group members and feeling understood, sparking a collective experience. Relatedly, listening to group member’s stories provided perspective on their own experiences, which served as a reminder that they were not alone in their suffering or that their suffering was relative to that of others. **3) Psychosocial benefit of intervention = easing pain;** adaptive response was a positive outcome from this group therapy and physiotherapy intervention |
| **EB** |  |  |  | increased tolerance for ambiguity | wider range of responses choices re relationships | recognise used to do x now d y | need for more information | sum bigger than parts | negotiation | recognises interactions between broken/ disrupted structures/ systems (political terror, war, forced migration) and adaptive/ maladaptive reactions among individuals/ groups | interactions with environment affects individual/ group reactions - maladaptive -> adaptive |
| **LB** | [Ponguta et al](https://onlinelibrary.wiley.com/doi/pdf/10.1002/cad.20314) | 2019 | **Fight**: harsh disciplinary styles by parents associated with poor child developmental and school readiness outcomes = several mothers reported yelling, depriving of privileges, ignoring, and hitting. At the same time, some mothers also reported at baseline using dialogue, speaking nicely, and applying corporal punishment as a last resort. | **Enablers to joining MOCEP program**: 1) Enabling and supportive reactions included openness and interest in new content and acceptability of exploring parenting strategies: *“People who are close to me encouraged me and said it is a good idea. Even if we are educated there are still things and skills that we don’t know so it is good to learn them from someone with experience.” 2) M*others expressed interest in learning new approaches to parenting—with an emphasis on effective strategies to discipline their children—as well as in promoting their own and their children’s social skills. | no info | Temporal changes: **1) Disciplinary practices improved:** After the intervention, disciplinary practices were largely focused on listening, dialogue, and redirecting behaviour. Corporal punishment and yelling were reported at endpoint to be used less frequently, compared to frequencies reported at baseline. After the intervention, mothers also reported changes in their own social and communication skills, as well as in their own feelings of resilience (e.g., ability to cope with stress) and wellbeing. With regard to perceived changes in the child, several mothers reported increased confidence in their children. **2) Perceived social cohesion didn’t change significantly:** there was a sense that the groups provided support, a space for socialization, and a vehicle to improve their self-confidence. However, there was no direct evidence to suggest the program had an impact on the general sense of security and community cohesion beyond the group meetings. **3) New positive relationships with trainers and other mothers:** Mothers also consistently noted a positive relationship with the trainers, across all sites, and, in many instances, the mothers established groups in social networking and communication apps/programs (e.g., WhatsApp) so that they could continue to connect with one another after the study ended. The statistical significance of the changes across these parenting domains and behaviours were quantitatively assessed in the outcomes of the RCT, and, as noted above, the results were much more promising among those mothers who participated in 14 or more of the 25 sessions (Ponguta et al., under review). **4) Fathers’ Reported Impact of MOCEP:** Changes in father’s engagement and interaction with children, Changes in mother’s interaction with father and children, Changes in mother’s interaction with other people outside the family. Fathers felt the program increased how assertive mothers where in terms of child-rearing practices, and they also noted an increase in social relationships with other women upon engaging in the program activities. | If they do something different maybe the other will do something different: Father's reported impact of MOCEP includes changes in interaction between father and mother = “Our relationship has become better, she’s in a better mood. Once she’s calm, I become calm so yes our lives are better than before.” – “She has more awareness. She has an ordered way of doing things, calculated, calm, she has a goal to work towards in the school. There have been good things. She teaches them better; she learns things and applies them on the children.” | no info | no info | 1) We were interested in documenting the process of implementing MOCEP—a program that targets parenting practices and knowledge with the aim to boost children’s holistic development and school readiness—in Lebanon. This is important because exposure to adversity during early childhood has been shown to be predictive of post-traumatic stress disorder among Syrian refugee children living in Lebanon (Karam et al., 2019). Caregiver support has been identified as a necessary, core component of humanitarian responses, yet parenting programs in these contexts are scarce (Cobham & Newnham, 2018). 2) A characterization of context should also be applied to determine ways in which existing practices, beliefs, and talents of the mothers can be included to sustainably empower the communities, while working in partnership with the mothers and families themselves to recognize their own potential and capital. 3) Key challenges to evaluations of ECD programs in fragile contexts include safety, security, and mobility; staff capacity and interest; and funding (Murphy et al., 2018). Our experience aligned well with the challenges reported in the existing literature, particularly with regard to safety and funding concerns. Another significant challenge to the evaluation of MOCEP was the application of an RCT design that would be acceptable to the community and would not affect the trust that mothers had in the community leaders, while maintaining scientific rigor. We found that the wait-list control design was acceptable to community leaders and to the communities themselves, but this was only achieved through extensive communication with mothers about the reason for the wait-list design and by operationalizing the successful retention of the wait-list control group. | 1) from intro: parental practices, such as harsh disciplinary style, have been associated with poor developmental and school readiness outcomes (Kilgore, Snyder, & Lentz, 2000). This is due, at least in part, to the negative effects of harsh parenting on the child’s emotional development and self-regulation which, in turn, influence other developmental outcomes and markers of school readiness. 2- **Father's reported impact of MOCEP** reflects improved emotional regulation (adaptive responses) of mothers via being calm and organised, which improves partner and family dynamics (bonds/networks). 3- RCTs in settings like the one described here are challenging. However, our research indicates that they are feasible if thoughtfully aligned with cultural and programmatic contexts. Moreover, they are critically important, along with implementation evaluations, in efforts to strengthen programs and to identify avenues for promoting empowerment and facilitating cohesion among families. |
| **EB** |  |  | in parenting |  | wider range of response choices when disciplining | awareness of what did prior to intervention and what do now, or other changes over time (pre/ post course) | The program had a positive impact on self-reported disciplinary practices of the mothers as well as on their level of self-reported parental stress. These findings were most robust for mothers who attended 14 or more of the 25 training sessions. A full presentation of the results is reported elsewhere (Ponguta et al., under review). After the intervention, disciplinary practices were largely focused on listening, dialogue, and redirecting behaviour. Corporal punishment and yelling were reported at endpoint to be used less frequently, compared to frequencies reported at baseline. After the intervention, mothers also reported changes in their own social and communication skills, as well as in their own feelings of resilience (e.g., ability to cope with stress) and wellbeing. With regard to perceived changes in the child, several mothers reported increased confidence in their children. fathers pointed to positive changes in their own parental practices as well as in their own relationship with the mothers. Other emergent themes with respect to the perceived changes in children were their self-confidence and social skills, as reported by the mothers in the FGDs. | in a sense had to withhold judgement to attend the course - was it just going to tell them how to parent or was there more to it; perhaps this is true of participation in all interventions | There were three emerging themes about the perceived impact of the program on mothers, as reported by the fathers. These included changes in fathers’ engagement and interactions with children, changes in mothers’ interactions with fathers and children, and changes in mothers’ interactions with other people outside the family (Table 5.4). In general, fathers felt the program increased how assertive mothers where in terms of child-rearing practices, and they also noted an increase in social relationships with other women upon engaging in the program activities. | contextualised | acknowledged role of environment (structures/ systems) as influenced parenting and mother-father relationship and wider community relationships (e.g., arrival of more refugees) |
| **LB** | [Eskici et al](https://pubmed.ncbi.nlm.nih.gov/34618479/) | 2021 | **Freeze**: Mental health disorders demonstrate a higher prevalence rate among refugees (Davis & Davis, 2006), and the most prevalent mental health problems are PTSD, anxiety, and depression. The World Health Organization (WHO) estimates that in conflict areas that the prevalence of PTSD is 15.3% and that of depression is 10.8%. | no info | no info | Temporal changes: 2- The effect sizes demonstrated that CA-CBT was more effective as compared to TAU in terms of symptom severity change, with nearly medium effect size for the HSCL-25 (d = .40) and very large effect size for HTQ (d = 1.17). | no info | no info | no info | Looking at existing systems like poverty, settings outside protection camps where MHPSS services are unavailable, cultural concepts of distress and gender-based experiences = **1) Many studies indicated that therapists who work with refugees should understand how the culture shapes people’s treatment expectancies, idioms of distress, experiences, and explanations of mental health** (Chung & Singer, 1995) and should implement therapeutic interventions that are compatible with the population’s worldview, beliefs, and culture (Bemak & Chung, 2014; Hinton & Patel, 2017). For refugees, language barriers, stigmatization, and lack of information about psychological disorders and treatment options are listed as barriers to access to mental health services (Morris et al., 2009). **2) Few studies have been conducted with Syrians living in Turkey and most of these studies were conducted in temporary protection camps.** Syrians who live outside of the temporary protection camps have more limited access to mental health services compared to Syrians in camps (Agbaba, 2016). Part of the cultural adaptation of CA-CBT is asking patients if they have culturally salient complaints (complaints that are common in a group, which may be, for example, certain somatic complaints or culture-specific expressions), addressing catastrophic cognitions about them, and giving positive expectancy that the treatment will help those complaints. This study was conducted with Syrian women who live outside of the camps. In addition, migration and poverty afflict Syrian women severely with studies demonstrating refugee women as a high-risk group. The current study focused on Syrian women, including taking gender-based traumatic experiences during war and immigration into consideration. Last, it is important to evaluate the effectiveness of CA-CBT due to its distinctive features that make it appropriate for this group, and the current study demonstrates acceptability and effectiveness. | CA-CBT intervention reduced maladaptive responses (anxiety, depression, PTSD, and # traumatic experiences) and found to be acceptable and feasible in a population of Syrian women who are high-risk and living outside camps with less access to MHPSS services: One of the important findings obtained in this RCT is that the CA-CBT was well accepted by Syrians, which is a group that has negative attitude toward mental health interventions. The feasibility and acceptability of the treatment is illustrated by the low drop-out rates and the absence of adverse events. Scalability is suggested by such features as group format and ease of administration. This current study also demonstrated CA-CBT’s large effect sizes for main outcomes. |
| **EB** |  |  |  |  |  |  |  |  |  | Mental health treatment costs, stigmatization, and the belief that mental health symptoms may get better in time seemed to be the main barriers to seeking treatment among Syrian refugees in Turkey (Fuhr et al., 2019). Related to structural (e.g., language barrier) and attitudinal barriers (e.g., stigma) there is a large mental health treatment gap among Syrian refugees in Istanbul, with a recent study indicating that less than 10% of Syrian refugees with mental health problems seek psychological help (Fuhr et al., 2019). | |
